# Predicting sepsis severity at first clinical presentation: The role of endotypes and mechanistic signatures

**DOI:** 10.1016/j.ebiom.2021.103776

**Published:** 2022-01-10

**Authors:** Arjun Baghela, Olga M. Pena, Amy H. Lee, Beverlie Baquir, Reza Falsafi, Andy An, Susan W. Farmer, Andrew Hurlburt, Alvaro Mondragon-Cardona, Juan Diego Rivera, Andrew Baker, Uriel Trahtemberg, Maryam Shojaei, Carlos Eduardo Jimenez-Canizales, Claudia C. dos Santos, Benjamin Tang, Hjalmar R. Bouma, Gabriela V. Cohen Freue, Robert E.W. Hancock

**Affiliations:** a Centre for Microbial Diseases and Immunity Research, University of British Colombia, 232-2259 Lower Mall, Vancouver V6T 1Z4, Canada; b Bioinformatics Graduate Program, Genome Sciences Centre, 570 W 7th Ave, Vancouver V5T 4S6, Canada; c Department of Molecular Biology and Biochemistry, Simon Fraser University, 8888 University Drive, Burnaby, B.C. V5A 1S6, Canada; d Vancouver General Hospital, 899 W 12th Ave, Vancouver V5Z 1M9, Canada; e Hospital Universitario Hernando Moncaleano, Calle 9 No. 15-25, Neiva, Colombia; f Department of Internal Medicine, Universidad Surcolombiana, Calle 9 Carrera 14, Neiva, Colombia; g Keenan Research Centre for Biomedical Science, Critical Care Medicine, St. Michael's Hospital, University of Toronto, 30 Bond Street, Toronto, ON M5G1W8, Canada; h The Westmead Institute for Medical Research, 176 Hawkesbury Rd, Westmead, NSW 2145, Australia; i Department of Internal Medicine, University Medical Center Groningen, University of Groningen, Hanzeplein 1, Groningen 9713 AV, the Netherland; j Department of Clinical Pharmacy and Pharmacology, University Medical Center Groningen, University of Groningen, Hanzeplein 1, Groningen 9713 AV, the Netherland; k Department of Statistics, University of British Columbia, 2207 Main Mall, Vancouver V6T 1Z4, Canada

**Keywords:** Sepsis, Severe sepsis, Endotypes, Gene signatures & biomarkers, Cellular reprogramming, Translational medicine

## Abstract

**Background:**

Inter-individual variability during sepsis limits appropriate triage of patients. Identifying, at first clinical presentation, gene expression signatures that predict subsequent severity will allow clinicians to identify the most at-risk groups of patients and enable appropriate antibiotic use.

**Methods:**

Blood RNA-Seq and clinical data were collected from 348 patients in four emergency rooms (ER) and one intensive-care-unit (ICU), and 44 healthy controls. Gene expression profiles were analyzed using machine learning and data mining to identify clinically relevant gene signatures reflecting disease severity, organ dysfunction, mortality, and specific endotypes/mechanisms.

**Findings:**

Gene expression signatures were obtained that predicted severity/organ dysfunction and mortality in both ER and ICU patients with accuracy/AUC of 77–80%. Network analysis revealed these signatures formed a coherent biological program, with specific but overlapping mechanisms/pathways. Given the heterogeneity of sepsis, we asked if patients could be assorted into discrete groups with distinct mechanisms (endotypes) and varying severity. Patients with early sepsis could be stratified into five distinct and novel mechanistic endotypes, named Neutrophilic-Suppressive/NPS, Inflammatory/INF, Innate-Host-Defense/IHD, Interferon/IFN, and Adaptive/ADA, each based on ∼200 unique gene expression differences, and distinct pathways/mechanisms (e.g., IL6/STAT3 in NPS). Endotypes had varying overall severity with two severe (NPS/INF) and one relatively benign (ADA) groupings, consistent with reanalysis of previous endotype studies. A 40 gene-classification tool (accuracy=96%) and several gene-pairs (accuracy=89–97%) accurately predicted endotype status in both ER and ICU validation cohorts.

**Interpretation:**

The severity and endotype signatures indicate that distinct immune signatures precede the onset of severe sepsis and lethality, providing a method to triage early sepsis patients.

**Research in context**

**Evidence before this study**

Sepsis is a common, life-threatening, and very heterogeneous condition; this has thwarted attempts at identification of high-risk patients, accurate early diagnosis, and development of disease-specific treatments. For every hour's delay in application of appropriate treatment (typically potent antibiotic regimens) there is a 76% increased risk of death. The issue of patient heterogeneity has been recently captured by separating patients into endotypes, where each endotype is subtype (phenotypic cluster) of sepsis, that is likely defined by a distinct functional or pathobiological mechanism. To identify studies in which severity had been analyzed or for which patient heterogeneity had been considered, in PubMed, we used the search terms (“sepsis” OR “septic shock” OR “severe sepsis”) AND (“gene expression profiling” OR “microarray” OR “RNA-Seq”) AND (“endotype” OR “cluster” OR “subgroup”); 36 studies described the use of gene expression profiling to examine the underlying molecular responses in infection and sepsis. We found that attempts to classify patients according to severity had to date been quite unsuccessful, in part because the rather inaccurate method of microarrays had been used to classify patients; critically only one study addressed pre-sepsis diagnosis patients from the ER. Thus, ten ICU studies used peripheral blood, microarray data and statistical methods to identify (usually two) endotypes in critically ill ICU patients with sepsis. Patients who were severely ill or died were generally scattered across all endotypes. Overall, these studies indicated that patients exhibiting reprogrammed or depressed immune systems generally experience higher clinical severity and increased mortality. It is expected this body of research will only grow in light of the global Covid-19 pandemic, since patients with severe SARS-CoV-2 infections and all-cause sepsis display overlapping molecular characteristics.

**Added value of this study**

Early prediction of severity and more accurate characterization of endotypes would allow physicians to apply, in a timely manner, the appropriate and most potent treatments in the critical early phase of sepsis. We investigated, prior to any formal diagnosis, 266 prospective sepsis patients in the ER from 4 continents using the highly accurate transcriptomics method, RNA-Seq. When combined with another cohort of 82 early ICU patients, we were able to identify small gene expression signatures that accurately predicted disease severity and mortality and were applicable to both early ER and early ICU patients. These signatures revealed related but distinct underlying mechanisms. To analyze the role of patient heterogeneity, we utilized the whole-blood gene expression data from our 266 ER patients, and machine learning test/validation procedures, to predict five distinct endotypes that captured the early sepsis response, within two hours of ER admission. These endotypes were defined by unique sets of ∼200 genes each, and distinct biological mechanisms and clinical outcomes, with two of the five endotypes associated with greater severity and mortality and one demonstrating much lower severity. We developed a classification model to accurately predict endotype status in two independent validation cohorts and two external datasets (from prior endotype studies), based on quantification of expression of 40 genes in whole blood; gene pairs from this classification subset were also able to accurately identify these endotypes. The endotypes were stable since 4/5 were also evident in ICU patients from our own and the two external datasets. Thus, early gene signatures of severity and endotypes can be identified from blood samples taken in the ER, and are relevant to patients regardless of disease stage, infection origin or location.

**Implications of all the available evidence**

This study demonstrates that the mechanisms relevant to the development of life-threatening sepsis can be observed as early as first entry into the ER, prior to any formal diagnosis. These include both discrete cross-cutting severity signatures and distinct endotypes associated with the propensity to develop severe disease. This considerably advances previous findings, indicating that sepsis patients display a high degree of heterogeneity, with a less certain relationship between severity, lethality and endotype. Indeed, reanalysis of these data demonstrated that patients previously proposed to fit into 2 or 4 endotypes could be reclassified into our 5 endotypes with similar severity profiles. This has important implications for sepsis patients and caregivers globally and suggests that the development of diagnostics and targeted therapeutics should focus on patients early in the sepsis disease course.

Alt-text: Unlabelled box

## Introduction

Sepsis is defined by a dysfunctional, life-threatening response to infection leading to (multi-)organ dysfunction and failure. The global burden is high, with an estimated 48.9 million cases of sepsis worldwide in 2017, leading to 11 million deaths, nearly 1 in 5 global deaths.^1^ Inter-individual clinical variability and lack of predictive and prognostic markers hinders efficient triage and expedient initiation of definitive therapy^2^,^3^ Furthermore, treatment delays impact strongly on early and late morbidity/mortality,^4^ while inappropriate antibiotic use has been linked to emergent resistance.^5^

Sepsis patients exhibit a very broad spectrum of disease manifestation, ranging from relatively mild to requiring ventilation, with varied organ dysfunction, septic shock, and death. Accordingly, given the clinical variability among sepsis patients there is only moderate consensus on how to accurately define the syndrome, especially at first clinical presentation. Blood transcriptomics has proven useful in obtaining systems-level descriptions of the responses dysregulated during sepsis. Gene expression signatures can to some extent discriminate between sepsis/acute infection and systemic inflammatory response syndrome (SIRS) (or “non-sepsis”) in the intensive care unit (ICU).^6^, ^7^, ^8^ However, these approaches typically lack sensitivity due to heterogeneity arising from individual genetic variation, demographic factors, the infection source and agent, therapeutic intervention, comorbidities including pre-existing immune-suppressive conditions, epigenetics, etc.^9^, ^10^, ^11^

These factors have led to sepsis being reframed as a syndrome comprised of several subgroups termed endotypes, which represent distinct biologically driven and clinically relevant groups of patients with varied severity and clinical outcomes^9^,^12^ Specifically, endotypes can provide more sensitive markers enabling prediction of sepsis severity, risk-stratification, and opportunities for individualized therapies. Previous research has concluded that 2–4 subgroups/endotypes exist in seriously ill sepsis patients in the ICU, that is after sepsis has been confirmed.^12^, ^13^, ^14^, ^15^ Results to date, largely driven by analysis of patient metadata and microarray transcriptomic studies, have indicated a single ICU specific endotype with higher severity scores, but patients with severe sepsis/lethality were scattered across endotypes. Although severe sepsis is no longer recognized as such in the Sepsis-3 definitions, we use it here to discriminate between severely ill patients and those with relatively mild disease. Moreover, the clinical utility of gene expression signatures identified in ICU patients are arguably less useful since patients have already deteriorated^3^, ^4^, ^5^ and require intensive care and antibiotics. If we were able to extend these analyses to identify severity markers and/or endotype status within the first hours of ER admission, this would enable more timely, aggressive and/or immunomodulatory interventions to prevent the further progression to more severe sepsis, while sparing broad-spectrum antibiotics when not needed.

Our objective was to identify novel transcriptional diagnostic and risk stratification markers at first presentation to the ER and ICU, when patients show less definitive/non-specific clinical traits, and sepsis diagnosis has not yet been established. We recruited a global cohort of patients from the Netherlands, Colombia, Canada, and Australia and used whole blood RNA-Seq and machine learning to develop gene expression signatures reflecting sepsis severity, cellular reprogramming, and mortality, as well as predicting 5 endotypes differing in overall severity. We exploited protein-protein interaction (PPI) networks to define mechanisms that collectively mediate these groupings. Severity markers and endotypes were further shown to be relevant to an independent prospective cohort of critically ill, tertiary-care ICU patients indicating that early gene expression endotypes were stable and associated with sepsis severity and mortality regardless of progression.

## Methods

### Study design and clinical data collection

In this study, we sought to characterize early molecular responses which dictate progression to severe sepsis. Accordingly, we enrolled diverse cohorts of adult patients (>18 years of age) with suspected sepsis from five hospital cohorts. ER patients were recruited if sepsis was suspected within 2 h of ER admission. This was based on the attending physician's informed opinion and required patients showing at least two SIRS/Sepsis-1 criteria^16^ and suspected infection. In some cases, at ER admission patients met the Sepsis-3 criteria^3^ for sepsis (SOFA scores >2), but since infection was not confirmed these patients were considered suspected sepsis. ICU patients with suspected pulmonary sepsis were enrolled prospectively in the COLOBILI study and generally recruited within of the first day of ICU admission. Patients were excluded if death was impending (within 12 h), if blood collection was unattainable, or consent was withheld. Enrollment included a full spectrum of individuals who might be suspected of being pre-septic. While we were aware of the possibility that early therapy might strongly influence outcomes for such patients, we made no attempt to correct for treatments that might influence outcome measures, since we were interested in the underlying mechanisms. To the best our knowledge, patients were not using any anti-inflammatories or steroids on the first day of ER and ICU admission. In total, we collected ER and ICU patients from five cohorts (Figure S1 and Table S3). There were 75 samples recruited from Colombia, 105 samples from the Netherlands, 13 from Vancouver (sample collection limited by the COVID-19 pandemic), 82 from Toronto, and 88 from Sydney. To enable retrospective association with gene expression data, various clinical and demographic metadata were collected at triage and within the 72 h following ER and ICU admission (Table 1). In addition, healthy control samples from either presurgical controls or healthy volunteers were obtained from Sydney, Vancouver, and Neiva (*n* = 44; average age 50 ± 24).

**Table 1:** **Sepsis severity and outcomes of patients included in the discovery and validation cohorts.** The mean value ± standard error is presented for numerical variables (total observations in brackets). Categorical variables are presented as % positive observations (relative to total observations). ER = value in the Emergency Room at first clinical presentation.

Parameter	Demographic and Clinical Data	
	ER Patients (*N* = 182 in Discovery group; 84 in Validation cohort)	ICU Patients (*N* = 82)
Age	56 ± 1·3 (266)	61·7 ± 1·7 (82)
Sex, Female	45·5% (121/266)	30·5% (25/82)
Location(s)	**Endotype Discovery:** Groningen, the Netherlands (39%); Neiva, Colombia (25%); Vancouver, Canada (4%); **Endotype Validation:** Sydney, Australia (32%)	Toronto, Canada (100%)
Duration of illness before ER/ICU arrival	6·1 ± 0·87 (266)	6·1 ± 0·85 (62)
ER qSOFA	1·0 ± 0·05 (266)	Not applicable
ER/ICU 24H SOFA Score	2·0 ± 0·12 (266)	7·2 ± 0·55 (79)
Hospital/ICU stay (days)	7·5 ± 0·54 (266)	12·0 ± 1·0 (82)
Blood Culture Result	19·2% (50/260)	12·2% (10/82)
ICU Admission	10·2% (27/266)	98% (80/82)*
Mortality (In hospital)	12·1% (32/265)	24·4% (20/82)
SARS-CoV-2 PCR Positive	None	32·9% (27/82)#

⁎ 2 patients collected from ward

# Based on PCR for SARS-CoV-2 RNA of nasopharyngeal and/or endotracheal tube aspirates. All patients had evident early sepsis.

### Ethics

All patients were enrolled under local ethical board approval. Informed written consent was obtained upon enrollment from the patient or their legal representative. The Clinical Research Ethics Board of the University of British Columbia (UBC) provided ethics approval for all sequencing and bioinformatics studies, carried out in a manner blinded to patient identity (approval number H17-01208).

### Blood collection and RNA-Seq

Three millilitres of blood was collected into PAXgene Blood RNA tubes (BD Biosciences) during usual blood collection to ensure intracellular RNA stabilization. RNA isolation, sample preparation, RNA-Seq and downstream processing was performed using well-established standard operating procedures.^17^ Total RNA was extracted using PAXgene Blood RNA Kit (Qiagen; Germantown, MD, USA). Quantification and quality measures of total RNA were obtained using an Agilent 2100 Bioanalyzer (Agilent; Santa Clara, USA). Poly-adenylated RNA was captured using NEBNext Poly(A) mRNA Magnetic Isolation Module (NEB; Ipswich, USA). cDNA libraries were prepared using the KAPA Total RNA HyperPrep Kit (Roche; Basel, Switzerland). RNA-Seq was then performed on Illumina Hi-Seq instrument using single read runs of 150 base-pair long sequence reads (excluding adapter/index sequences). RNA-Seq processing and count matrix generation are described in the Supplemental Materials. Of the 363 patients recruited, 348 showed RINs > 65 and had sequencing libraries greater than 1 million (Australia/84, Colombia/67, the Netherlands/104, Vancouver/11, Toronto/82). Severity analysis used all patients, while endotype classification utilized 182 patients in the ER discovery set, 84 patients in the Sydney ER validation cohort and 82 patients in the Toronto ICU validation cohort (Figure S1). Raw sequencing data are available at NCBI GEO (Accession Number GSE185263).

### Statistics

Mechanisms associated with severity were identified from all patients (ER+ICU) using differential expression (DE) analysis with DESeq2,^18^ followed by over-representation/enrichment of up- and down-regulated genes using the Reactome^19^ pathway and MSigDB Hallmark^20^ databases. Patient severity was assessed using Sequential Organ Failure Assessment (SOFA) scores,^3^ a proxy for organ dysfunction, sepsis, and mortality risk. We developed signatures for markers of severity groups and mortality by using logistic regression with least absolute shrinkage and selection operator (LASSO).^21^ The severity signatures were obtained by comparing patients with extreme phenotypes (i.e., High vs Low severity groups), whereby input to the LASSO regression model included up and down-regulated genes from the lists of DE genes between these groups. We hypothesized that the extreme severity group phenotypes would yield the most robust markers of severity. The LASSO-selected severity signature was then assessed for predictive power by comparing the High + Intermediate vs Low severity groups. The mortality signature was obtained similarly, with input to the LASSO model being DE genes between Dead vs Surviving patients. We also validated the use of a previously published cellular-reprogramming (CR)^22^,^23^ signature of immune suppression using the random forest algorithm.

An unsupervised machine-learning method, *k-*medoids consensus clustering, was applied to detect endotypes with similar gene expression profiles. In preliminary studies, we attempted various clustering methods including *k*-medoids clustering, Density-Based Spatial Clustering of Application with Noise (DBSCAN), and *K*-means clustering and employed different distance metrics. All clustering methods largely yielded the same results, except for approximately 7% of patients who clustered differently between methods. We chose to cluster gene expression profiles with *k*-medoids clustering (with Manhattan distance) since this method is robust to outlier samples and features that can arise from high throughput sequencing.^24^ The appropriate number of clusters/endotypes (*k* = 5) was determined by applying cluster validity metrics (Table S4; Figure S2). To understand the mechanistic underpinnings of endotypes, we extracted DE genes and associated pathways, by comparison to healthy controls and to each other. Cell proportions were estimated using CIBERSORT,^25^ and correlated to measured cell counts where available. Clinical parameters were compared between endotypes using Kruskal-Wallis and Chi-Squared tests to compare numerical and categorical variables, respectively. Survival analysis was performed using Kaplan Meir estimation with log-rank tests. Five unique endotype signatures were obtained by identifying the 200 most-upregulated DE genes that were unique to each endotype (comparing each endotype to all others). These signatures enabled a minimized 40-gene endotype classification/prediction tool using multinomial regression model with LASSO regularization. To further reduce the genes required for endotype classification, we determined pairs of genes from the 40-gene multinomial model that could reliably stratify patients into endotypes (Supplemental Methods). The multinomial model was then used to assign endotype status among patients of the validation cohorts.

### Role of funders

The funders (Canadian Institutes for Health Research; FDN-154287) had no role in the study's design, data collection and analysis, interpretation, or writing of the manuscript.

## Results

### Severity, mortality, and cellular reprogramming display overlapping mechanisms

To identify markers of sepsis severity in sepsis patients at first clinical presentation, a multi-country whole-blood RNA-Seq study was performed. ER patients (266; Table 1) from 4 countries/continents and early ICU patients (82) demonstrated the strong within-cohort heterogeneity, for which sepsis as a syndrome is well known.^9^ To capture the full range of severity, all 348 ER and ICU patients were included in the analysis, capturing the full spectrum of early septic individuals. For ER patients, the average age was 56 ± 13 years (range 19–96) and average 24 h post admission SOFA scores were 2·0 ± 0·12 (range 0–10), with an average hospital stay of 7·5 ± 0·54 days (range 1–65). In-hospital mortality was moderate at 12·1%, cf. the global mortality of 23% in sepsis,^1^ consistent with the concept^23^ that around 50% of patients with early suspected sepsis subsequently develop more severe sepsis. In contrast, the ICU cohort displayed much higher severity; the average age was 61·7 ± 1·7, the average SOFA score was 7·2 ± 0·55 (24H post- admission), and the rates of mortality were much higher at 24·4%.

**Figure 1:**
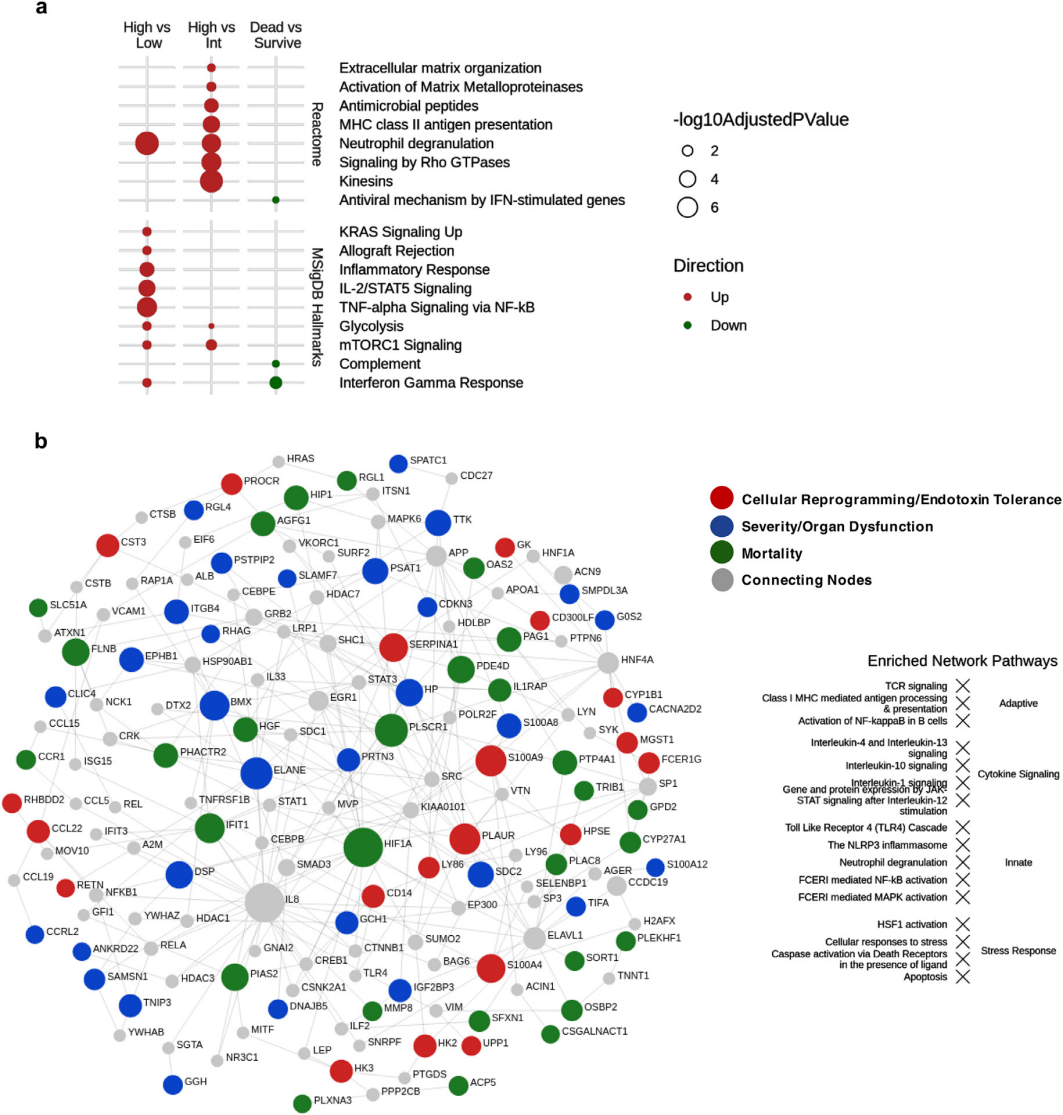
**Biological characterization of SOFA-based severity groups and mortality in all ER and ICU patients.** (a) Functional enrichment of up and downregulated DE genes (displaying ≥ ±1.25-fold change; adjusted *p* ≤ 0·05) comparing severity and mortality. SOFA scores were dichotomized into High (*n* = 82), Intermediate (*n* = 125), and Low (*n* = 138) groups. Functional characterization of DE genes was performed using an overrepresentation analysis of Reactome pathways or MSigDB sets (adjusted *p*-value≤0·05). (b) A combined PPI network (drawn using NetworkAnalyst^45^) of the severity (52 genes/proteins), mortality (38 genes/proteins), and cellular reprogramming (31 genes) biological signatures. PPI represent function-based interactions in cells. Thus the formation of a cohesive network indicated that the products of the genes involved are functionally related and collectively regulate or play key roles in one or more related biological mechanisms. The nodes (coloured circles) represent the signature genes and the edges (lines connecting the nodes) represent a curated (i.e. known) interaction between the genes (or rather their protein equivalents). The size of the node represents its connectivity (i.e. how many other proteins it interconnects with), whereby highly connected genes (or hubs) are larger. The mortality signature shared no genes in common with the severity signature but shared one gene with the CR signature (DHRS9). The severity signature shared three genes in common with the CR signature (PSTPIP2, RAB13, S100A12).

We explored whether there were gene expression markers of severity and mortality when considering patients who progressed to High (24 h SOFA scores ≥5), Intermediate (SOFA ≥2; <5), or Low (SOFA <2) severity. We also explored potential markers of in-hospital mortality (Figure 1). Comparing High vs. Intermediate and High vs Low severity groups (representing the extreme phenotypes) yielded several upregulated immune-related processes (Figure 1a), particularly neutrophil degranulation, the most enriched pathway when comparing severity groups. This indicated sepsis severity, at least in the early stages, is associated with neutrophil activity. When comparing patients who died in the hospital to those that survived, the interferon-γ response was notably downregulated. This was consistent with the concept that Interferon-γ is a key mediator of post-sepsis immunosuppression while Interferon-γ therapy can improve patient prognosis.^26^,^27^

Using LASSO regression, two mechanistic signatures were derived (Table S1) comprised of DE genes from the High vs. Low and Dead vs. Surviving comparisons and showed good AUCs (equated to accuracy) of 70–80% (Table 3), and significant statistical enrichment in ER patients (Table S2). A distinct CR signature of severe sepsis, and an 8-gene sub-signature, had similar cross validation AUCs/Accuracy of 75–77%, and similar levels of sensitivity and specificity (Table 3). Importantly, a recent meta-analysis of qSOFA scores, which is measured at ER admission, in fact showed quite poor ability to predict severity/mortality. Specifically, qSOFA scores show sensitivity of ∼42%,^28^ which our signatures substantially outperformed. To understand the mechanisms unifying these signatures, we created function-based protein-protein interaction (PPI) networks. Interestingly, the severity, CR and mortality signatures could be integrated into a single coherent network (Figure 1b). This indicated that the mechanisms related to each were discrete but biologically related since well-connected networks reflect complementary and/or inter-connected gene expression responses and pathways. Network hubs (highly interconnected nodes) included ELANE and CYP19A1 (severity signature); PLAUR, S100A9, SERPINA1, and HK2 (CR signature); HIF1A, PLSCR1, and IFIT1 (mortality signature). Furthermore, STAT1/3, ELAV1, SP1, IL8/33, TLR4, and JUN were first-order interactors that connected together the signature genes nodes.

### Stratification of ER patients with suspicion of sepsis into five mechanistic endotypes

Comparing severity groups indicated the relationship between the clinically defined groups and gene expression was good but not excellent which we ascribed to patient/sepsis heterogeneity. Previous studies demonstrated an uncertain relationship between severity and endotype.^13^, ^14^, ^15^ Here we characterized sepsis endotypes using our more accurate RNA-Seq dataset to observe if this was an additional factor determining severity. In particular we identified endotypes in suspected sepsis patients at first clinical presentation, where timely immunomodulatory interventions would be most effective in preventing further progression.

**Figure 2:**
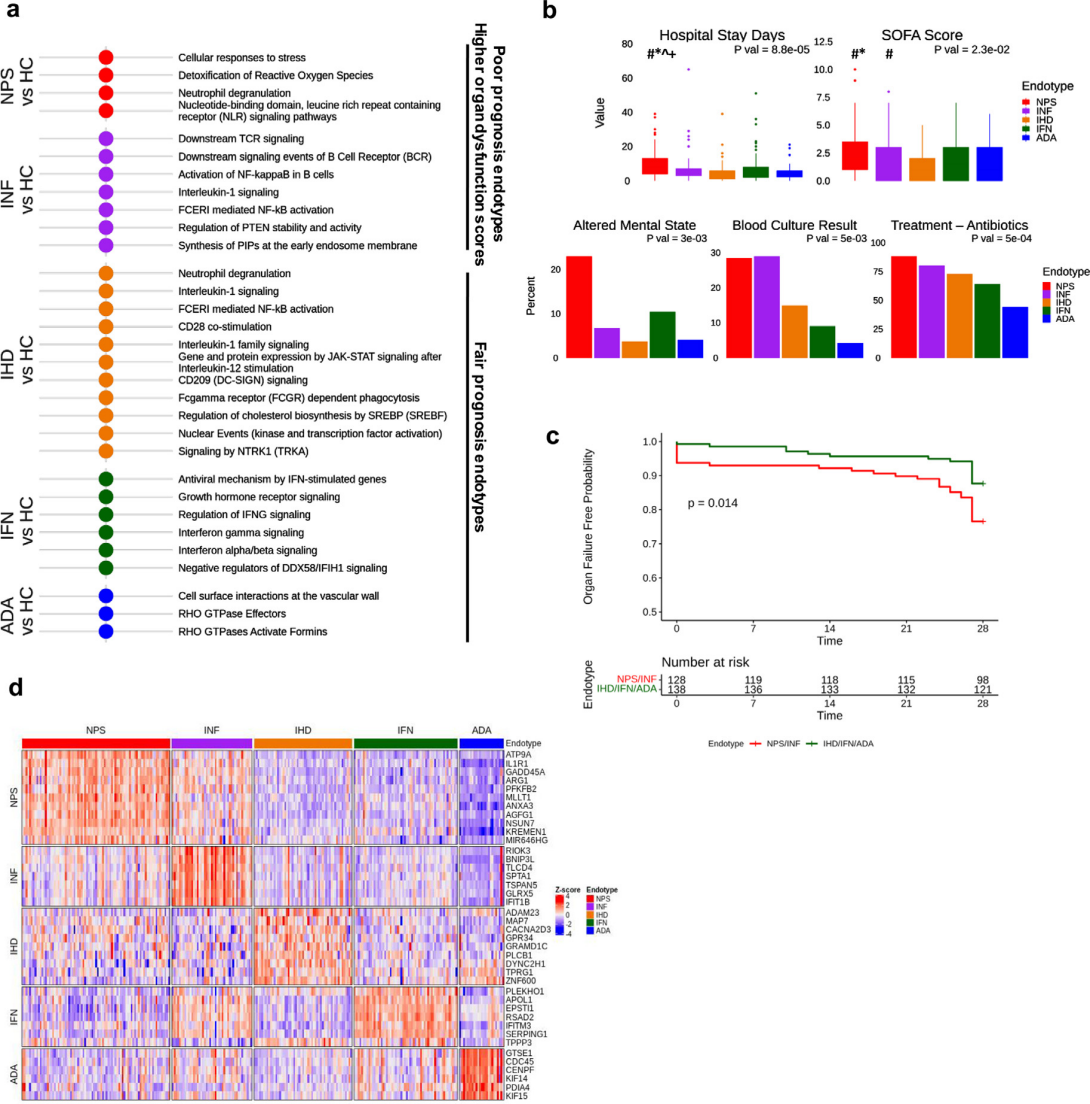
**Biological and clinical characterization of Neutrophilic-Suppressive (NPS), Inflammatory (INF), Innate-Host-Defence (IHD), Interferon (IFN), and Adaptive ADA) endotypes and their respective signatures.** (a) Functional enrichment of up and downregulated DE genes (displaying ≥ ±1.5-fold change; adjusted *p* ≤ 0.05) comparing each endotype to healthy controls (*n* = 39). (b) Selected clinical symptomology and outcomes of endotypes and their distributions. Dunn's Posthoc test indicated by: # *p* < 0·05 cf. IHD; * *p* < 0·05 cf. IFN; + *p* < 0·05 cf. ADA; ^ *p* < 0·05 cf. INF. (c) Kaplan-Meier curves describing 28-day organ failure free days. Organ failure free days was compared between endotypes by combining the low prognosis endotypes (NPS and INF) and the fair prognosis endotypes (IFN, IHD, and ADA). The combined endotypes shared many molecular and clinical features, so this scheme made biological sense and increased statistical power to detect a significant difference. (d) Heat map showing the expression of 40 classification genes (used to drive the endotype classification model) in all patients (arrayed left to right). NB this signature delivered excellent performance in the discovery group (AUC/accuracy: 96%; Sensitivity: 81%; Specificity: 95%).

Based on differential gene expression profiles, five endotypes were identified in a discovery cohort (*n* = 182) of ER patients from the Netherlands, Colombia, and Canada, and subsequently validated in a validation cohort (*n* = 84) from Australia (Table S3, Figs. S2,S3). Based on differentially expressed pathways/mechanisms, endotypes were denoted as Neutrophilic-Suppressive (NPS), Inflammatory (INF), Innate Host Defence (IHD), Interferon (IFN), and Adaptive (ADA) with diverse clinical parameters. Five distinct signatures of 182–200 upregulated genes (Table S6), reflecting coherent mechanisms, discriminated each endotype from all others. This enabled, using LASSO multinomial regression, the generation of a 40-gene classification gene-set for endotype prediction, with a very high AUC/Accuracy of 96% (Table 3). Subsequently we analyzed all 266 ER patients to improve the robustness of mechanistic and phenotypic conclusions (Figure 2).

### Five early sepsis endotypes were mechanistically diverse and varied in prognoses

**Figure 3:**
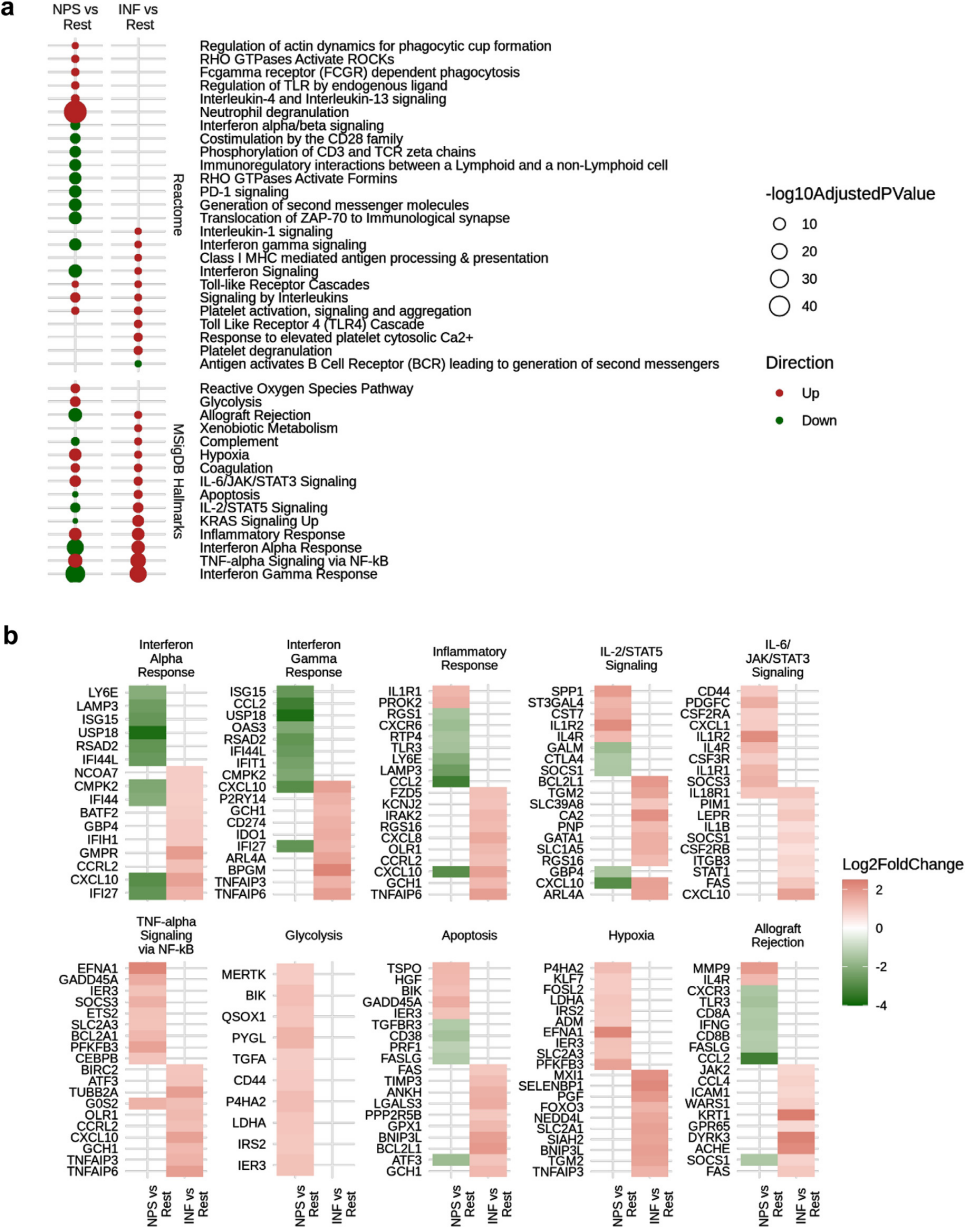
**Detailed mechanistic characterization of the poor prognosis NPS and INF endotypes.** (a) Functional enrichment of up- and down- regulated DE genes when comparing the NPS endotype to all other endotypes combined and the INF endotype to all others. (b) Fold changes of genes associated to NPS and INF related processes, which reflect potential mediators and regulators of the endotype. The NPS endotype is clearly immunosuppressed as indicated by the downregulation of several processes, including inflammation, interferon processes, and PD-1 signaling among others.

The spectrum of altered biological mechanisms for each endotype in the ER patients was characterized by comparing the unique ∼200-gene signatures for each endotype relative to a set of 39 healthy controls (Figure 2a). These endotype signatures revealed distinct signalling pathways and processes mediating each endotype. The NPS endotype involved upregulated genes in several innate immune system pathways, particularly neutrophil degranulation, responses to stress, NLR pathways, and reactive oxygen species production, and downregulation of adaptive immune and interferon signalling pathways (Figures. 2a, S5). The INF endotype was most similar to the NPS endotype but demonstrated strong differences including increased inflammatory responses, NFκB activation, and activation of TCR/BCR signaling (Figures. 2a, 3). Analysis of cell types by estimates using CIBERSORT and, where possible (229 patients) actual measurements, indicated that the NPS/INF endotypes had higher neutrophil proportions (Figure S5d). Except for the ADA endotype, proportions of five other immune cell subsets demonstrated minor (≤20%) changes between endotypes and healthy controls.

The IFN, ADA and IHD endotypes showed overlapping gene expression, based on principal component analysis, but also substantial differences (Figures. 2, S5). The IFN endotype showed high expression of interferon-α, -β, and -γ signaling processes. The ADA endotype was notable for Rho GTPases, mTORC1 signaling and glycolysis, and upregulation of adaptive immune pathways, and more abundant lymphocytes (Figs. S3, S5). The IHD endotype showed the fewest DE genes reflecting innate host defences, including moderate upregulation of interleukin signalling.

Importantly, the endotypes were linked to substantial differences in clinical parameters and severity outcomes (Table 2, Figure 2b, c). On average, the NPS and INF endotypes were clearly associated with more severe disease based on organ failure probability (log-rank *p* = 0.014), 24 h SOFA-scores (Kruskal-Wallis *p* = 0·0022), 72 h SOFA-scores (Kruskal-Wallis *p* = 0·038), hospital stay days (Kruskal-Wallis *p* = 0·00019), requirement for O_2_ therapy (Chi-Squared *p* = 0·019), and assessment of a positive blood culture (Chi-squared *p* = 0·001). However individual patients in each endotype had broadly different outcomes that might be explained in part by the timeliness of appropriate treatment and other unknown variables. The NPS endotype displayed the longest hospital stays, consistent with early evidence of CR and immunosuppression that might hinder rapid resolution.

**Table 2:** **Clinical data of patients belonging to endotypes in combined (discovery and validation) analysis.** The mean value ± standard error is presented for numerical variables with the total available observations/ patient numbers recorded in brackets. Categorical variables are presented as percent positive (% total positive/total available observations). *P* values are derived from Kruskal-Wallis and Chi squared tests testing for significant differences between endotypes for numerical and categorical values, respectively. Not included are readmission within 6 months, ER systolic, ER temperature, ICU admission, mortality, ER respiratory rate, ER urea, ER creatinine, and gender that were not significantly different (*p* > 0·05) between groups.

Parameter	Mechanistic Endotypes					
	NPS (*N* = 83)	INF (*N* = 45)	IHD (*N* = 55)	IFN (*N* = 58)	ADA (*N* = 25)	*P* Val
Age (years)	59·4 ± 2·17 (83)	58·3 ± 2·59 (45)	62·7 ± 2·52 (55)	51·2 ± 2·73 (58)	37·2 ± 3·93 (25)	*p* < 0·0001
Hospital Stay Days	9·6 ± 0·9 (81)	7·8 ± 1·6 (45)	4·9 ±0·88 (54)	7·6 ± 1·26 (58)	5·3 ± 1·25 (22)	*p* < 0·0001
ER Temperature (C)	37·6 ± 0·16 (81)	38·2 ± 0·18 (45)	37·4 ± 0·13 (55)	38·1 ± 0·14 (58)	37·3 ± 0·22	0·00014
ER Systolic (mm Hg)	115·7 ± 2·47 (83)	121·6 ± 3·44 (45)	131·9 ± 3·21 (55)	118·9 ± 2·87 (57)	111·2 ± 3·44 (25)	0·00030
ER MAP^a^ (mm Hg)	90·5 ± 2·2 (83)	94·3 ± 2·9 (45)	105 ± 2·9 (55)	92·7 ± 2·7 (57)	89 ± 3·2 (25)	0·00069
ER Heart Rate (beats/ min)	104·2 ± 2·5 (83)	110·3 ± 2·7 (45)	97·4 ± 2·7 (55)	109 ± 2·5 (57)	95·8 ±4·2 (25)	0·00094
At ER Altered Mental State	22·9% (19/83)	6·7% (3/45)	3·6% (2/55)	10·3% (6/58)	4% (1/25)	0·0030
Treatment – Antibiotics	88% (73/83)	80% (36/45)	72·7% (40/55)	63·8% (37/58)	44% (11/25)	0·0050
Blood Culture Result	28·4% (23/81)	28·9% (13/45)	14·8% (8/54)	8·9% (5/56)	4·2% (1/24)	0·0050
At ER qSOFA	1·2 ± 0·09 (83)	0·9 ± 0·12 (45)	0·7 ± 0·08 (55)	0·9 ± 0·1 (58)	1·2 ± 0·18 (25)	0·0094
ER Urea	10·7 ± 0·85 (81)	7·6 ± 0·79 (44)	11 ± 103 (52)	8·8 ± 0·92 (55)	9·1 ± 0·94 (23)	0·014
Treatment - O_2_ Therapy	41% (34/83)	37·8% (17/45)	29·1% (16/55)	26·3% (15/57)	8% (2/25)	0·019
ER 24H SOFA Score	2·5 ± 0·24 (83)	2·2 ± 0·32 (45)	1·4 ± 0·21 (55)	1·8 ± 0·24 (58)	1·7 ± 0·34 (25)	0·023
ER Creatinine	106 ± 519 (83)	89·2 ± 7·9 (44)	113 ± 897 (55)	108 ± 132 (56)	80·5 ± 4·37 (24)	0·026
ER Diastolic (mm Hg)	70·3 ± 1·6 (81)	68·5 ± 2·3 (45)	75·6 ± 1·9 (55)	73·5 ± 1·8 (57)	67·6 ± 1·8 (25)	0·046
Within 72 h SOFA	1·5 ± 0·24 (83)	1·8 ± 0·41 (45)	0·5 ± 0·15 (55)	1·6 ± 0·36 (58)	1 ± 0·31 (25)	0·060

a MAP = Mean arterial pressure

The 40-gene classification set derived from the unique gene-sets for each endotype revealed distinct expression patterns for each endotype in ER patients (Figure 2d), with endotype-specific genes generally downregulated in the other endotypes. These 40 genes facilitated the derivation of 148 gene pairs in the ER discovery set that were able to separately predict each of the 5 endotypes with AUCs/Accuracy as high as 89–97% (Tables 3, S7), indicating high diagnostic potential.

**Table 3:** **Model performance statistics for endotype predictions and outcome measures predicting impending severity.** The AUC (≡ Accuracy), sensitivity, and specificity of the models, expressed as percent is provided. The performance of the 40 gene signature and the top 5/148 performing gene pairs is shown. For the severity comparisons, comparisons are based on patient groups with SOFA scores measured 24 H post ER/ICU admission: High (≥ 5), Intermediate (≥ 2 and < 5), and Low (< 2). The cellular reprogramming/endotoxin tolerance (CR) Signature is as per Pena et al.^23^ The severity and mortality signatures were further reduced by filtering the genes input to LASSO using a more stringent fold change cut off (top 25% highest fold changes).

		Cross Validation (%)		
Comparison	Gene Sets	AUC ≡ Accuracy	Sensitivity	Specificity
**Severity**^a^				
High vs. Low Severity	Severity Signature	80	72	73
	Reduced Severity Signature^c^	80	76	70
	CR Signature	75	70	68
	Reduced CR Signature^d^	77	73	73
High + Intermediate vs. Low Severity	Severity Signature	71	72	60
	Reduced Severity Signature^c^	69	64	63
	CR Signature	68	64	62
	Reduced CR Signature^d^	73	67	67
Survived vs Died	Mortality	75	68	70
	Reduced Mortality Signature^e^	67	62	62
	CR Signature	60	57	61
	Reduced CR Signature^c^	62	60	61
**Endotypes**^b^				
Multinomial Model	40 gene set	96	81	95
NPS vs. all others	MLLT1/NSUN7	97	93	91
INF vs. all others	SPTA1/GLRX5	95	90	85
IHD vs. all others	MAP7/PLCB1	90	85	79
IFN vs. all others	PLEKHO1/EPSTI1	86	83	76
ADA vs. all others	CENPF/PDIA4	97	87	91

a AUCs obtained in the full (ER + ICU) discovery cohorts.

b AUCs of data-driven endotype gene sets obtained in ER discovery cohort.

c Reduced Severity Signature = TNIP3, DSP, RHAG, G0S2, ITGB4, GPR84, FAM83A, PCOLCE2, CXCL8, SDC2, PRTN3, ELANE

d Reduced CR signature = CD300LF, CPVL, CST3, HK3, MGST1, RAB13, RETN, S100A12

e Reduced Mortality Signature = HGF, DHRS9, SIGLEC1, MS4A4A, OAS2, MMP8, RGL1, SLC51A, OSBP2, IFIT1

### The NPS endotype captured the most severe and immunosuppressed patients

Poorer prognosis, elevated Neutrophil proportions, and down-regulation of certain inflammatory markers and adaptive signaling pathways indicated that the NPS endotype tended to feature nascent immunosuppression. Downregulated pathways included downregulated CD28 co-stimulation, CD3/TCR phosphorylation, immunoregulatory lymphoid and non-lymphoid cell interactions, PD1 signalling, interferon-γ signalling and Zap-70 translocation to immunological synapses (Figure 3a). Conversely the other severe endotype INF led to upregulation of multiple inflammatory pathways. These differences could be explained in part by differential expression of elements of signalling pathways such as IL2/STAT5, IL-6/JAK/STAT3, Interferon, and TNF-α/NFκB (Figure 3b) For example, based on *in vivo* knockout models,^29^ STAT3 signaling is known to induce anti-inflammatory and immune-suppressive conditions in immune cells.

### ICU patients retained severe prognosis endotypes

The presence of these mechanistically and clinically relevant sepsis endotypes was validated in a sub-cohort of 82 critically ill patients (Table 1) enrolled in the COLOBILI study (St. Michael's Hospital, Toronto). Patients had severe respiratory failure and suspected pulmonary sepsis on day-1 of ICU admission. This ICU cohort demonstrated higher severity and poorer outcomes cf. the ER cohorts (Table 1).

**Figure 4:**
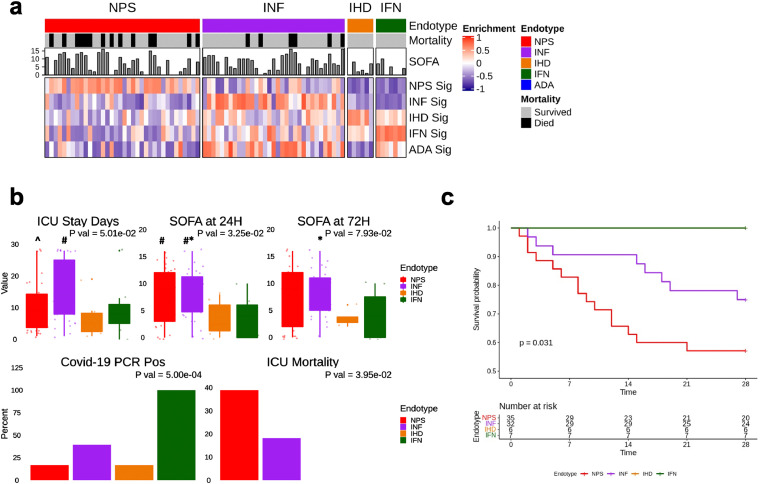
**Endotype classification of ICU patients.** (a) Heatmap depicting GSVA enrichment statistics in ICU patients (*n* = 82) for each endotype signature. Each 200-gene endotype was significantly upregulated in the patients classified to the endotype for which it defines. (b) Selected clinical symptomology and outcomes for predicted endotypes; Dunn's Posthoc test indicated by: # *p* < 0.05 cf. IHD; * *p* < 0·05 cf. IFN; ^ *p* < 0·05 cf. INF. (c) Kaplan-Meier curves describing 28-day mortality.

The endotype classifier was applied to predict endotype status (Figure 4a) and Gene-Set Variation Analysis (GSVA)^30^ was used to measure the enrichment of the five endotype signatures. This classified ICU patients into 4 endotypes with most (84%) fitting into the more severe NPS/INF endotypes. The ADA endotype was not identified, consistent with the observed and opposite downregulation of immune processes in later-stage sepsis patients. The NPS and INF endotypes showed higher severity with higher 24 h SOFA scores (mean 7.9; Kruskal-Wallis *p* = 0·033) (Figure 4b; Table 4, S10) and significantly higher lethality, with the NPS endotype displaying significantly higher 28-day mortality (45·7%) cf. the INF endotype (25·9%). Intriguingly, none of the 13 patients assigned to the IFN or IHD endotypes died. The 7 ICU patients classified as belonging to the IFN endotype were all COVID-19 positive, suggesting a potential association to viral infections. Nevertheless, while all ICU patients demonstrating the IFN endotype had COVID-19, this was only 25% of COVID patients studied. The remainder of the COVID-19 patients fit the general patterns observed for other ICU patients, with those that died fitting into the more severe and deadly NPS and INF endotypes. We also assessed the enrichment of our endotype signatures in the ICU expression (microarray) data of the Davenport et al.^14^ (SRS1/2) and Scicluna et al.^15^ (Mars1-4) endotypes (Figure S6). This indicated that ICU patients could be re-classified based on the distinct mechanisms we uncovered.

**Table 4:** **Severity and outcomes of the endotypes in the ICU cohort.** The mean value ± standard error is presented for numerical variables with the total available observations/ patient numbers recorded in brackets. Categorical variables are presented as percent positive (% total positive/total available observations). *P* values are derived from Kruskal-Wallis and Chi squared tests testing for significant differences between endotypes for numerical and categorical values, respectively.

	Mechanistic Endotypes				
Parameter	NPH (*N* = 36)	INF (*N* = 33)	IHD (*N* = 6)	IFN (*N* = 7)	*P* Val
Covid-19 PCR Positivity	16·7% (6/36)	39·4% (13/33)	16·7% (1/6)	100% (7/7)	0·00050
Mortality within 28 Days	45·7% (16/35)	25·9% (7/27)	0% (0/5)	0% (0/6)	0·025
SOFA 24H post ICU admission	7·6 ± 0·9 (34)	8·2 ± 0·78 (32)	3·5 ± 1·34 (6)	3·7 ± 1·49 (7)	0·033
ICU Mortality	38·9% (14/36)	18·2% (6/33)	0% (0/6)	0% (0/5)	0·034
ICU Stay Days	10·4 ± 1·29 (36)	15·2 ± 1·63 (33)	6·8 ± 2·7 (6)	9·7 ± 3·43 (7)	0·050
SOFA 48H post admission	7·5 ± 0·98 (31)	8·4 ± 0·75 (30)	3·5 ± 0·87 (4)	4·1 ± 1·7 (7)	0·079
SOFA at ICU admission	8·4 ± 0·9 (36)	7·9 ± 0·64 (33)	4·2 ± 1·7 (6)	5 ± 1·66 (7)	0·093

The ICU cohort analysis demonstrated that endotypes persisted during deterioration of patients to severe sepsis. To eliminate the possibility that the endotypes were sorting patients according to just critical illness/severity, we obtained publicly available peripheral blood gene expression data (RNA-Seq) from patients with non-infectious/sterile myocardial infarction (GSE103182).^31^ The endotype assignment of this cohort (93.3% IHD and 6·7% IFN) did not show association with the authors’ original comparison of interest (i.e., ST-elevated myocardial infarction/STEMI vs non-ST-elevated myocardial infarction/NSTEMI; Chi-squared *p* = 1·0). Similarly, endotype assignment of an unpublished cohort of cancer patients with acute infection (50·0% INF, 33·3% IHD, and 16·7% ADA) did not show any association with cancer type, namely Leukemia or Lymphoma (Chi-square *p* = 0·89). These results suggest the endotypes, particularly the NPS and INF endotypes, did not only reflect severe illness and captured mechanisms distinct from other conditions.

## Discussion

Sepsis is notorious for the clinical heterogeneity observed in patients, who often demonstrate broad, non-specific symptomatology in the ER, but can rapidly deteriorate thereafter. It is generally accepted by clinicians that each hour's delay in initiating appropriate therapy costs lives, with early potent antibiotics being applied to dampen underlying infection in the hope that sepsis progression is hindered.^4^,^32^ This has the downside of contributing to antimicrobial resistance, since broad-spectrum antibiotics are used even when there is no observable bacterial infection.^5^ In this study, we identified gene expression signatures that can be assessed in patients as early as 1,2 h after ER admission. The signatures can be rapidly measured in hospitals (using PCR, a method that is freely available in most hospital labs) after routine blood collection from prospective sepsis patients). Future studies will be required to enable assessment of these signatures on an appropriate (likely multiplex qRT-PCR) platform before this clinical potential is realized. Assessment of these signatures would provide additional input for physicians to triage patients, in addition to currently used criteria such as lactate, qSOFA, C-reactive protein, Glasgow Coma score and various SIRS assessments.

Several single mRNA-based biomarkers have been proposed to predict sepsis status in patients including the S100 family of genes, C Reactive Protein (CRP) and various pro- and anti-inflammatory cytokines (e.g., IL-10, IL-6, IL-12, TNF)^11^,^33^ However, these single biomarkers have showed nominal prognostic accuracy, reflecting their inability to capture a holistic view of the complex immune responses involved in sepsis,^34^ and critically have not been validated in very early sepsis. Thus, they are often correlative rather than predictive. Accordingly, various groups have exploited whole-blood gene expression microarrays to identify, in ICU patients, multi-gene signatures predictive of sepsis; far fewer studies have correlated this with impending deterioration and severity.^6^, ^7^, ^8^^,^^34^,^35^ The Pediatric PERSEVERE model, identified a biomarker set to estimate the probability of mortality at the time of admission.^35^ In adults, Septicyte Lab^36^ and the FAIM3:PLAC8 ratio^8^ discriminated infected from healthy controls, showing diagnostic accuracy analogous to C-reactive protein,^13^ but discrimination from non-infectious inflammation was less encouraging. All three approaches performed well at identifying survivors, but, unlike the current study, poorly at identifying non-survivors and distinct subgroups varying in morbidity. Intriguingly, the previously elucidated CR severe-sepsis signature as well as DE severity-related and mortality-related gene-sets, could be assembled into cohesive pathways and protein: protein interaction networks, unifying their underlying mechanisms into a single biological program. This fact, together with the mechanistically driven endotype signatures, underpins the potential value of new diagnostics to guide physician decision-making as well as new mechanism-based personalized medicine approaches.

Our study indicates that endotypes are independent determinants of severity/mortality. Endotypes are clinically relevant subgroups of a condition, wherein each endotype is defined by distinct biological mechanisms. Numerous clinical studies have failed to identify individual biomarkers specific to sepsis,^37^ likely because the presence of heterogeneous subgroups was not considered. Identification of reliable endotypes in sepsis, particularly in its earliest stages, enabled us to dissect the heterogeneous molecular responses at play and provided prognostic and severity signatures. Using the largest prospective, observational, and blinded (to patient identity and clinical data) RNA-Seq omics studies performed to date on possible sepsis patients at first clinical presentation, we determined that patients could be assorted into 5 distinct endotypes, four of which were retained in ICU patients, cf. previous studies suggesting only 2 endotypes in the ICU. Our 5 endotypes were characterized by distinct gene expression profiles and signatures, novel mechanistic underpinnings, and different but overlapping clinical factors, including severity. Critically they were identified in an ER discovery group from three countries/continents and confirmed in a fourth independent cohort and in ICU patients. We also predicted our endotypes in patients of the Davenport et al.^14^ (SRS1/2) and Scicluna et al.^15^ (Mars1-4) endotypes (Figure S6). Intriguingly, despite the limitations of using microarray data, the highest lethality groups in the ICU in these studies (SRS1 and Mars1) aligned best with the NPS and INF endotypes respectively, perhaps reflecting differences in selection of patient populations.

The NPS and INF endotypes displayed higher estimated neutrophil proportions, cf. our other endotypes, and clustered to some extent on PCA, but maintained significant differences including >200 unique DE genes. These endotypes, detected in ER patients, were associated with higher subsequent SOFA scores, longer hospital stays, and a 2·7-fold increase in positive blood culture results, among other clinical parameters, and intriguingly were the most prominent endotypes preserved in ICU patients. STAT3 was a connecting node in the CR/severity/mortality network and intriguingly the IL6/STAT3 signaling pathway was notably upregulated in the NPS endotype signature. This suggested a regulatory role of this pathway in sepsis and severe illness, and potentially an early molecule in immunosuppression. The higher neutrophil proportions observed in these endotypes might also be associated with a tendency towards severe outcomes. Neutrophils have a paradoxical role in sepsis, including their potentially-beneficial role in first-line host defences against microbes, contrasting with their contribution to organ dysfunction of over-stimulated or reprogrammed neutrophils.^38^ Indeed, elevated neutrophil to lymphocyte ratios are associated with poor outcomes in sepsis patients.^39^ Conversely, sepsis-induced neutrophil dysfunction is associated with increased risk of nosocomial and secondary infections, consistent the more severe symptomology and outcomes of the NPS and INF endotypes.^40^

The endotypes provide a substantial opportunity for personalized care and identify different mechanisms that predict or are associated with severe sepsis, and thus can be targeted. The clear immunosuppression associated with the NPS endotype would suggest early application of immune recovery therapies (e.g., interferon gamma therapy, Granulocyte-Macrophage Colony Stimulating Factor/GM-CSF, interleukin-7/IL-7).^41^ The inflammatory profile of the INF endotype identifies a group of patients who might improve with targeted anti-inflammatory therapies. Interestingly, based on clinical features many studies have uncovered subgroups based on clinical features, termed sub-phenotypes, which appear to display overlap in the symptomatology and outcomes with the NPS and INF endotypes^42^, ^43^, ^44^ The IFN, IHD, and ADA endotypes would suggest close monitoring without immediate administration of antibiotics, potentially decreasing the overuse of antibiotics. Intriguingly, severity/mortality signatures, endotypes and underlying mechanisms were clearly conserved between early sepsis in the ER and ICU patients. Taken together, these data reveal that early sepsis signatures are applicable to both a wide variety of ER patients as well as severely ill patients, at the first day of ICU admission.

## Contributors

RH and OP conceived the study. AB, AL, OP, SF and RH contributed to the study design. AB and AL performed bioinformatics analysis. AB and RH drafted the manuscript. AB, AHL, BB, AA, OP, RH contributed to interpretation of data. AB, GCF, and RH contributed to statistical analysis. BT, HB, CdS, CJ, AH, and UT coordinated, and AM, JR, AB, MS, and CJ were directly involved in, sample and patient metadata collection in hospitals. All authors edited the manuscript. RF processed samples for sequencing. AB, OP, AL, BB, AA verified the quality and accuracy of sequencing and clinical data. RH was responsible for obtaining funding and led the study. All authors have read and approved the final version of the manuscript.

## Data sharing

Raw RNA sequencing data and de-identified patient metadata (including sex, age, clinical severity measures, and outcomes) is available at NCBI GEO (Accession Number GSE185263). Other materials, including study protocol, statistical analysis plan, and code is available to anyone who requests. These requests should be directed to REW Hancock (bob@hancocklab.com).

## Declaration of interests

RH has filed the CR and endotype signatures for patent protection and licenced this to Sepset Biotherapeutics Inc., a Vancouver company in which he has a significant ownership position. AB and GCF have filed the endotype signatures for patent protection and licenced this to Sepset Biotherapeutics Inc. Other authors have nothing to disclose.

## Appendix Supplementary materials

Image, application 1

Image, application 2
