# Supplementary material for: Predicting sepsis severity at first clinical presentation: The role of endotypes and mechanistic signatures
Source: eBioMedicine. 2022 Jan 10;75:103776. doi: 10.1016/j.ebiom.2021.103776 (PMC8808161; doi:10.1016/j.ebiom.2021.103776)
Supplement: Supplementary file 1 [file mmc1.docx]

**SUPPLEMENTAL MATERIALS**

***Supplementary Figures***

**Figure S1. Outline of patient collection and analyses.** (a) We enrolled adult patients (> 18 years of age) using local ethical approvals, from four ERs and one ICU. . ER patients were recruited if sepsis was suspected within 2 hours of ER admission. This was based on the attending physician’s informed opinion, but nonetheless required patients showing at least two SIRS/Sepsis-1 criteria and suspected infection. In some cases, at ER admission patients also showed SOFA scores >2 (therefore meeting the Sepsis-3 criteria for sepsis), but since infection was not confirmed these patients were still considered suspected sepsis. ICU patients were recruited with suspicion of pulmonary sepsis with and/or without COVID-19. These ICU patients were enrolled prospectively in the COVID19 Longitudinal Biomarkers of Lung Injury (COLOBILI) study and recruited within the first day of ICU admission (with the exception of two patients who were recruited from the hospital ward). Of these, 27 were confirmed to be infected with SARS-CoV-2 by subsequent viral PCR. Enrollment included a full spectrum of individuals who might be suspected of being pre-septic, and we made no attempt to correct for treatments that might influence outcome measures since we were interested in the underlying mechanisms. To our knowledge, patients were not using any anti-inflammatories or steroids at the first day of ER and ICU admission. Available medical records indicated patients displayed infections with diverse origins, including influenza virus, dengue virus, COVID-19, Escherichia coli, and Methicillin-resistant Staphylococcus aureus (MRSA). (b) Specific cohorts used to discover and validate signatures. There were 75 samples recruited from Colombia, 105 samples from Netherlands, 13 from Vancouver, 82 from Toronto, and 88 from Sydney. Of these 363 patients, 348 showed RNA integrity numbers (RINs) > 6.5 and had sequencing libraries greater than 1 million reads (84/Australia, 67/Colombia, 104 Netherlands, 11 Vancouver). Healthy control samples were either recruited locally in Vancouver at UBC (according to UBC ethics approvals) or were presurgical patients. They ranged in age from 20-80 years old (average: 46). (c) The endotype analysis was used to identify data-driven patient groups, which we denoted as the Neutrophilic Suppressive (NPS), Inflammatory (INF), Innate Host Defences (IHD), Interferon (IFN), and Adaptive (ADA). Their biological/clinical character was assessed by comparison of gene expression profiles to healthy controls and to each other. The severity analysis was used to characterize the gene expression profiles of clinically relevant severity groups. Specifically, SOFA scores were dichotomized into High (SOFA ≥ 5), Intermediate (SOFA ≥ 2 and <5 ), and Low (SOFA < 2) groups. When comparing endotypes to healthy controls, and clinically defined severity groups/endpoints (i.e. SOFA severity groups, and in-hospital mortality) using DE analysis, cell proportions were included in the linear model to correct for their contribution to fold change. Separate sets of healthy controls were used to compare endotypes of the discovery and validation cohorts.

**Figure S2**

**Figure S2. Consensus matrix and cumulative distribution function (CDF) plots from consensus clustering of the top 5% most variable genes matrix in the discovery set (n = 182).** (a) Consensus k-medoids clustering was used to cluster patients based on gene expression profiles. Consensus clustering, also referred to as ensemble clustering, is an algorithm that performs repeated clustering on subsamples of a portion of samples and genes. K-medoids with Manhattan distance was used for clustering since it is more robust to outliers, which are common in high-throughput omics data, when compared to other methods such as hierarchical clustering. Consensus clustering provided a consensus of the repeated clustering, which was robust to sampling variability. The consensus was represented as a consensus matrix, where each element is the fraction of times two samples that clustered together (a perfectly stable consensus matrix would consist of a matrix of 1’s and 0’s). Consensus heatmaps indicating cluster assignment when *k* was 2, 3, 4, and 5. Cluster assignment is indicated at the top of the heatmap. Dark blue indicates samples clustered with other samples within the cluster consistently over repetitions (b) Consensus CDF for *k* values from 2 to 10. The flatness of the curve is an indication of clustering stability. (c) Elbow plot displaying the relative change in the area under the CDF (AUCDF) curve for *k* values from 2 to 10.

**Figure S3. Biological characterization of Inflammatory, Neutrophilic-Suppressive, Interferon, Adaptive, and Innate Host Defence endotypes in the discovery cohort (n = 182).** (a) Functional enrichment of each ~200-gene endotype signature (182 genes only for the IFN endotype). The endotype signatures were derived by comparing each endotype to all others. Each endotype signature is mediated by unique signaling pathways and mechanisms, highlighting the diversity in sepsis endotypes captured at first clinical presentation. (b) Heatmap depicting endotype status and SOFA scores for each patient in the discovery cohort. Includes GSVA enrichment statistics for each endotype signature. (c) Heatmap depicting expression of the 40-gene mechanistic endotype classification set with respect to endotypes in the discovery cohort. (d) AUCROC curves for the top performing diagnostic gene pairs for each endotype. (e) Heatmap depicting endotype status and SOFA scores for each patient in the ER validation cohort. (f) Heatmap depicting expression of the 40-gene mechanistic endotype classification set with respect to endotypes in the ER validation cohort.

**Figure S4. Minimum order protein:protein interaction networks of each 200 gene Endotype signature. (a-e).** Each 200 gene signature formed a well-connected minimally connected fist order network when drawn using NetworkAnalyst,^38^ indicating the genes involved are functionally related and collectively regulate or play key roles in one or more related biological mechanisms. Red nodes are genes with increased expression specific to the endotype while grey nodes are interconnecting first order interaction nodes. Lines represent edges which are known protein:protein interactions derived from www.innatedb.ca. The size of the nodes indicates the hub degree or how well interconnected any given node is to all other nodes in the Network interconnecting lines (where Hubs are key molecules in signalling since they are highly interconnected; they receive and integrate multiple signals and pass them on to downstream nodes). **(f)** Minimum order network of 40 gene multinomial signature indicates the LASSO selected genes are functionally diverse but still connect through first/second order interactions. Colour code: Red = NPS; Violet = INF, Orange = IHD; Green = IFN; Blue = ADA; Grey = First/second order interacting genes. The formation of a well-connected combined Network comprising 33 out of the 40 signature genes, indicates that these endotypes are influencing discrete mechanisms in processes that are interrelated.

**Figure S5. Biological characterization of Inflammatory, Neutrophilic-Suppressive, Interferon, Adaptive, and Innate Host Defence endotypes in the combined ER (discovery and validation) cohort.** (a) Principal component analysis indicated the separation of endotypes based on global gene expression profiles (n = 266). (b) Fold change of Pena *et al* CR (right) and inflammatory (left) genes for each endotype. (c) Cell proportions as estimated by CIBERSORT for each endotype. (d) Correlation of CIBERSORT^24^ estimated cell proportions and measured cell proportions. Differential cell counts were available for 229 patients. For the available data there was a moderate predictive relationship between measured and computed cell proportions. (e) Neutrophil to Lymphocyte ratios for each endotype. Ratios were available for 220 patients. Dunn’s Posthoc test: # p<0·05 cf. IHD; * p<0·05 cf. IFN; + p<0·05 cf. ADA; ^ p<0·05 cf. INF.

**Figure S6**

**Figure S6. Endotype classification of ICU validation cohort and ICU patients of the Scicluna and Davenport endotypes.** (a) Heatmaps depicting expression of the 40-gene mechanistic endotype classification set in the ICU cohort (n = 82). (b) SRS1 and 2 microarray and metadata (n = 265) from Davenport et al^14^ was obtained from ArrayExpress (accession: E-MTAB-4421). The SRS1 endotype, which was associated with higher mortality compared to the SRS2 endotype, showed high expression of the NPS signature. The SRS2 endotype showed high expression of the IHD, IFN, or ADA signatures, but obviously did not further divide the endotype based on the mechanisms we discovered. (c) Mars1-4 expression and metadata (n = 479) from Scicluna et al^15^ was obtained from Gene Expression Omnibus (accession: GSE65682). The INF endotype signature was highly enriched in the Mars1 endotype, which was associated with the highest mortality compared to the other endotypes. Interestingly, Heme biosynthesis was a predominant molecular feature of Mars1 (also a feature of the INF endotype), which is an important concurrent feature of hyper-inflammatory phenotypes. The NPS endotype signature was enriched in the Mars2 endotype which displayed the highest severity scores and the highest rates of septic shock across discovery and validation cohorts. Consistent with our observations, Mars2 also showed significant overlap with SRS1.^14^ Taken together, it appears that our study and that of Davenport et al definitely overlap to some extent (although we identified 3 other endotypes), but do not entirely agree with Scicluna et al, although our reanalysis of their data shows much better alignment. The notable advance of our study is in the diversity of immune responses and clinical traits detected at ER admission, some of which may not be applicable to responses in severely ill ICU patients.

***Supplementary Tables***

**Table S1. Severity and Mortality signatures derived from the full ER and ICU cohorts (n = 348).** LASSO was used to refine the DE genes of the respective comparison to obtain a reduced set of predictive and biologically-relevant signatures. Genes in the signatures are indicated by bold typeface. There were 52 genes in the severity signature and 38 genes in the mortality signature.

| **Gene Name** | **Fold change** | |
| --- | --- | --- |
|  | **Non-survivors vs. Survivors** | **Severe (High) vs. non-severe (Low)** |
| HGF | **-1.51** | 1.34 |
| FAR2 | **-1.28** | -1.05 |
| DHRS9 | **-1.55** | 1.22 |
| PAG1 | **-1.27** | 1.2 |
| PIAS2 | **1.34** | -1.1 |
| SIGLEC1 | **-2.47** | -1.47 |
| HIF1A | **-1.27** | 1.19 |
| ACP5 | **1.49** | 1.08 |
| MS4A4A | **-1.52** | 1.27 |
| OAS2 | **-1.88** | -1.18 |
| PTP4A1 | **-1.31** | -1.07 |
| PHACTR2 | **-1.42** | 1.1 |
| PDE4D | **-1.39** | 1.29 |
| GPD2 | **-1.31** | 1.05 |
| MMP8 | **-2.28** | 2.02 |
| HIP1 | **-1.35** | 1.2 |
| PLXNA3 | **1.26** | -1.18 |
| SORT1 | **-1.32** | 1.26 |
| KCNH3 | **1.62** | -1.12 |
| CYP27A1 | **1.58** | 1.05 |
| FLNB | **-1.42** | 1.05 |
| RASGEF1B | **-1.49** | 1.04 |
| RGL1 | **-1.51** | 1.21 |
| PLAC8 | **-1.45** | 1.39 |
| CSGALNACT1 | **-1.39** | 1.08 |
| CCR1 | **-1.43** | -1.05 |
| SLC51A | **-1.86** | 2.09 |
| SFXN1 | **-1.4** | 1.22 |
| PLEKHF1 | **1.37** | -1.24 |
| TRIB1 | **-1.27** | 1.13 |
| PEAK1 | **-1.26** | 1.04 |
| AGFG1 | **-1.26** | 1.15 |
| OSBP2 | **2.15** | 1.31 |
| IFIT1 | **-2.13** | -1.1 |
| PLSCR1 | **-1.39** | 1.14 |
| IL1RAP | **-1.44** | -1.05 |
| SDHAF3 | **-1.3** | 1.16 |
| TTN-AS1 | **-1.28** | 1.09 |
| CACNA2D2 | 1.01 | **-1.55** |
| BAIAP3 | 1.34 | **-1.91** |
| SLAMF7 | 1.06 | **1.59** |
| TNIP3 | -1.53 | **2.84** |
| DSP | -1.83 | **-2.86** |
| CDKN3 | 1.01 | **1.81** |
| SLCO4A1 | -1.25 | **2.2** |
| BMX | -1.25 | **1.53** |
| CNTNAP3 | -1.08 | **-1.76** |
| RHAG | 1.43 | **2.37** |
| TTK | -1.18 | **1.96** |
| CCRL2 | 1.09 | **1.51** |
| G0S2 | -1.17 | **2.61** |
| ATP1B2 | 1.34 | **2.18** |
| GCH1 | -1.13 | **1.6** |
| SERPINF1 | 1.18 | **-1.66** |
| ITGB4 | 1.11 | **-2.1** |
| PSAT1 | -1.09 | **1.54** |
| IGF2BP3 | -1.24 | **1.56** |
| DNAJB5 | -1.01 | **-1.88** |
| GGH | -1.06 | **1.51** |
| SLC39A8 | -1.28 | **2.16** |
| GPR84 | -1.39 | **2.41** |
| MORN3 | -1 | **-1.6** |
| RAB13 | 1.01 | **1.57** |
| S100A8 | -1.16 | **1.55** |
| TIFA | 1 | **1.71** |
| FAM83A | 1.32 | **9.22** |
| PSTPIP2 | -1.11 | **1.52** |
| TCTEX1D1 | -1.69 | **2.21** |
| ANKRD22 | -1.15 | **2.09** |
| EPHB1 | -1.15 | **-1.69** |
| SAMSN1 | -1.36 | **1.53** |
| RGL4 | -1.2 | **1.54** |
| S100A12 | -1.27 | **1.68** |
| PCOLCE2 | -1.4 | **2.25** |
| CLEC4D | -1.29 | **1.54** |
| CXCL8 | -1.14 | **2.8** |
| SDC2 | -1.75 | **-2.24** |
| CLIC4 | -1.05 | **1.65** |
| SMPDL3A | -1.45 | **1.62** |
| TIGD3 | 1.23 | **-1.79** |
| ADGRE1 | -1.31 | **1.61** |
| GRAMD1C | -1.29 | **-1.78** |
| RELL1 | -1.05 | **-1.7** |
| FFAR3 | -1.62 | **1.9** |
| SPATC1 | 1.1 | **2.03** |
| PRTN3 | 1.42 | **2.85** |
| SLC28A3 | -1.41 | **1.85** |
| ELANE | 1.21 | **2.49** |
| CFAP45 | 1.2 | **-1.65** |
| HP | -1.16 | **1.86** |

**Table S2. Enrichment of the CR, Severity, and Mortality signatures in the ER and ICU cohorts separately.** In general, the severity signatures were more enriched in ER patients, indicating the signatures were applicable to patients at first clinical presentation, while the mortality signature was more enriched in the ICU patients. Statistically significant enrichment is indicated by bold typeface.

| **Cohort** | **Comparison** | **Statistical significance (p-value)** | | |
| --- | --- | --- | --- | --- |
|  |  | **CR** | **Severity** | **Mortality** |
| **ER** | High + Intermediate vs. Low | **0.010** | **0.0081** | **0.0010** |
|  | High vs. Low | 0.072 | **0.0014** | **0.0014** |
|  | High vs. Intermediate | 0.9298 | **0.027** | 0.10 |
|  | ICU Admission vs. Not | 0.16 | 0.45 | 0.21 |
|  | Dead vs. Survive | 0.50 | 0.39 | 0.24 |
| **ICU** | High + Intermediate vs. Low | 0.067 | 0.33 | 0.27 |
|  | High vs. Low | 0.083 | 0.12 | 0.16 |
|  | High vs. Intermediate | 0.99 | **0.0044** | 0.12 |
|  | Dead vs. Survive | **0.0033** | **0.0049** | **0.00010** |

**Table S3. Sepsis severity and outcomes of patients included in the ER endotype discovery and validation cohorts.** The mean value, standard deviation, and total observations used are presented for numerical variables, i.e. mean (±SD; total observations). Categorical variables are presented as total positive observations, percent, and total observations, i.e. total positives (percent; total observations). ER = value in the Emergency Room at first clinical presentation.

| **Parameter** | **ER Discovery Cohort (N= 182)** | **ER Validation Cohort (N= 84)** |
| --- | --- | --- |
| Age | 55.6 ± 1.55 (182) | 57 ± 2.18 (84) |
| Sex, Female | 42.9% (78/182) | 51.2% (43/84) |
| Location(s) | Groningen, Netherlands (57%); Neiva, Colombia (37%); Vancouver, Canada (6%) | Sydney, Australia (100%) |
| Duration of illness before ER/ICU arrival | 7 ± 1.22 (182) | 4 ± 0.71 (83) |
| ER qSOFA | 1 ± 0.06 (182) | 0.8 ± 0.09 (84) |
| ER/ICU 24H SOFA Score | 1.8 ± 0.14 (182) | 2.3 ± 0.24 (84) |
| At ER/ICU 72H SOFA | 1.1 ± 0.16 (182) | 1.8 ± 0.27 (84) |
| Hospital/ICU stay (days) | 8.1 ± 0.71 (176) | 6.3 ± 0.75 (84) |
| Blood Culture Result | 19% (34/179) | 19.8% (16/81) |
| ICU Admission | 9.3% (17/182) | 11.9% (10/84) |
| Mortality | 12.7% (23/181) | 10.7% (9/84) |

**Table S4. Optimal *k* values calculated using cluster validation metrics.** Determining the optimal number of subgroups or clusters in a given disease, or more generally a data matrix is complex. In the absence of extensive domain knowledge and to avoid subjective decision making, we employed various empirical validity metrics to determine the number of sepsis endotypes, including Gap Statistic,^51^ Silhouette,^52^ Connectivity,^53^ and consensus clustering cumulative distribution functions (CDF).^54^ We evaluated k values ranging from two to ten and applied validation metrics to determine the optimal number of clusters present. Further, to minimize the impact of noisy and irrelevant genes on the clustering results, we ranked genes by variance (mean absolute deviation) and performed cluster validation on the clustering inputs comprised of the top 5 to 100 percent of genes (examining the top 5%, 10%, 25%, 50%, 75%, and 100% of genes). Ultimately, the clustering input and *k* cluster value that were stable using the fewest input genes, were selected. We found that *k* cluster values of two and five were optimal using input genes for the top 5% to 100% of genes. The stability of these results indicated that using the top 5% of genes for clustering was appropriate. We opted to stratify the patient population beyond two clusters, thus selected five clusters for further characterization. Further, we avoided exploring 5+ clusters, as this would likely reduce the clinical relevance of each cluster.

| **Method** | **Top 5%** | **Top 10%** | **Top 25%** | **Top 50%** | **Top 75%** | **All genes** |
| --- | --- | --- | --- | --- | --- | --- |
| Consensus Cluster CDF | 5 | 5 | 5 | 5 | 5 | 5 |
| Gap Statistic | 5+ | 5+ | 5+ | 5+ | 5+ | 5+ |
| Silhouette/Connectivity | 2 | 2 | 2 | 2 | 2 | 2 |

**Table S5. Sepsis severity and outcomes of the Mechanistic endotypes in the discovery cohort.** The mean value, standard error, and total available observations for numerical variables, i.e. mean ± standard error (n). Categorical variables are presented as percent positive (positive/n), i.e. percent (total positive/total available observations). ER = value in the Emergency Room at first clinical presentation. P values derived from Kruskal-Wallis and Chi square testing for significant differences between endotypes. Abbreviations: NPS = Neutrophilic-Suppressive; INF = Inflammatory; IHD = Innate Host Defence; IFN = Interferon; ADA = Adaptive.

| **Parameter** | **Mechanistic Endotypes** | | | | | **P Value** |
| --- | --- | --- | --- | --- | --- | --- |
|  | **NPS (N= 58)** | **INF (N=26)** | **IHD (N=30)** | **IFN (N=47)** | **ADA (N=21)** |  |
| Age | 58 ± 2·83 (58) | 59·5 ± 3·09 (26) | 63·9 ± 3·06 (30) | 54·3 ± 2·95 (47) | 35·2 ± 4·23 (21) | < 0.0001 |
| Treatment – Antibiotics | 84·5% (49/58) | 84·6% (22/26) | 76·7% (23/30) | 53·2% (25/47) | 33·3% (7/21) | 0.00050 |
| ER Diastolic (mmHg) | 73·3 ± 1·99 (58) | 65·2 ± 2·42 (26) | 79·7 ± 2·34 (30) | 75·3 ± 2·17 (47) | 68·5 ± 1·95 (21) | 0·0010 |
| Treatment – Oxygen Therapy | 43·1% (25/58) | 53·8% (14/26) | 33·3% (10/30) | 19·1% (9/47) | 0% (0/21) | 0·0010 |
| Hospital Stay Days | 9·7 ± 1·27 (56) | 12·3 ± 2·81 (26) | 6·4 ± 1·5 (30) | 6·5 ± 0·98 (46) | 3·9 ± 1·07 (18) | 0·0014 |
| ER Systolic (mmHg) | 120·6 ± 3·06 (58) | 116 ± 3·85 (26) | 137·6 ± 3·97 (30) | 121·9 ± 3·32 (47) | 111·2 ± 4·01 (21) | 0.0015 |
| ER FIO2 (%) | 26·5 ± 1·85 (51) | 23·6 ± 1 (22) | 22·7 ± 1·03 (23) | 22 ± 0·53 (40) | 21 ± 0 (20) | 0·0023 |
| Blood Culture Result | 24·6% (14/57) | 38·5% (10/26) | 16·7% (5/30) | 10·9% (5/46) | 0% (0/20) | 0·0075 |
| ER Temperature  (Celsius) | 37·4 ± 0·2 (58) | 37·8 ± 0·21 (26) | 37 ± 0·16 (30) | 37·6 ± 0·15 (47) | 37·2 ± 0·25 (21) | 0·052 |
| ER MAP  (mmHg) | 99 ± 2·65 (58) | 95·1 ± 3·68 (26) | 111·1 ± 3·54 (30) | 100·9 ± 3·24 (47) | 91·4 ± 3·5 (21) | 0·013 |
| ER qSOFA | 1·2 ± 0·09 (58) | 1·2 ± 0·17 (26) | 0·8 ± 0·11 (30) | 0·9 ± 0·12 (47) | 1·3 ± 0·2 (21) | 0·026 |
| Within 72 SOFA | 1·2 ± 0·28 (58) | 2·4 ± 0·71 (26) | 0·4 ± 0·17 (30) | 0·8 ± 0·22 (47) | 0·9 ± 0·34 (21) | 0·084 |
| Readmit in ≤6 Month | 21·6% (11/51) | 33·3% (8/24) | 17·2% (5/29) | 11·1% (5/45) | 9·5% (2/21) | 0·1 |
| ER Altered Mental State | 19% (11/58) | 19·2% (5/26) | 3·3% (1/30) | 8·5% (4/47) | 4·8% (1/21) | 0·11 |
| ER Creatinine | 103·6 ± 6 (58) | 108·6 ± 25·34 (25) | 115·8 ± 11·92 (30) | 100·7 ± 9·57 (46) | 81 ± 4·37 (20) | 0·14 |
| ER Heart Rate (beats/min) | 102·6 ± 2·85 (58) | 107·9 ± 3·41 (26) | 98·9 ± 3·18 (30) | 100·4 ± 2·74 (47) | 93·7 ± 4·62 (21) | 0·21 |
| ER Urea (mmol/L) | 10·3 ± 0·8 (57) | 10·8 ± 1·97 (25) | 13·6 ± 1·52 (30) | 10·1 ± 1·08 (44) | 9·3 ± 0·91 (20) | 0·21 |
| ER SOFA Score | 2 ± 0·23 (58) | 2·5 ± 0·5 (26) | 1·7 ± 0·31 (30) | 1·4 ± 0·22 (47) | 1·7 ± 0·39 (21) | 0·27 |
| ICU Admission | 10·3% (6/58) | 19·2% (5/26) | 10% (3/30) | 4·3% (2/47) | 4·8% (1/21) | 0·29 |
| ER Respiratory Rate (breaths/ min) | 22·8 ± 0·86 (57) | 22·2 ± 1·36 (25) | 23·3 ± 1·05 (28) | 20·8 ± 0·65 (43) | 21 ± 0·64 (20) | 0·31 |
| Sex (% female) | 36·2% (21/58) | 53·8% (14/26) | 33·3% (10/30) | 46·8% (22/47) | 52·4% (11/21) | 0·33 |
| Mortality | 10·5% (6/57) | 23·1% (6/26) | 16·7% (5/30) | 10·6% (5/47) | 4·8% (1/21) | 0·34 |
| Duration of Illness Prior to ER Arrival | 7·1 ± 3·1 (58) | 8·6 ± 2·63 (26) | 6·4 ± 1·99 (30) | 7·4 ± 2 (47) | 5·1 ± 0·97 (21) | 0·47 |
| Antibiotics Prior to ER Arrival | 22·4% (13/58) | 38·5% (10/26) | 16·7% (5/30) | 27·7% (13/47) | 23·8% (5/21) | 0·62 |
| Readmit within 28 Days | 12·5% (7/56) | 8% (2/25) | 16·7% (5/30) | 15·2% (7/46) | 9·5% (2/21) | 0·87 |

**Table S6.** **Top 200 differentially expressed genes differentiating each endotype from each other endotype.**

This Table is provided as a separate Excel file.

**Table S7. Classification tool comprising differentially expressed gene collectively predictive of endotype status.** There were 40 genes selected from the lists provided as Table S6 using a multinomial regression model, selected in part due to high fold changes (FC) when comparing each endotype to all others using DE analysis.

| **Gene Name** | **Description** | **Fold Change** | | | | |
| --- | --- | --- | --- | --- | --- | --- |
|  |  | **NPS vs. Rest** | **INF vs. Rest** | **IHD vs. Rest** | **IFN vs. Rest** | **ADA vs. Rest** |
| NSUN7 | NOP2/Sun RNA methyltransferase FM7 | **4.57** | 1.21 | -2.82 | -3.36 | -10.61 |
| ATP9A | ATPase phospholipid transporting 9A | **4.16** | 1.46 | -3.59 | -4.31 | -4.14 |
| PFKFB2 | 6-phosphofructo-2-kinase 2 | **4.06** | 1.82 | -3.51 | -4.97 | -10.43 |
| ARG1 | arginase 1 | **3.83** | 1.63 | -2.16 | -5.8 | -9.04 |
| ANXA3 | annexin A3 | **3.55** | 1.72 | -3.84 | -2.77 | -12.15 |
| IL1R1 | interleukin 1 receptor type 1 | **3.28** | 1.26 | -1.8 | -2.58 | -8.64 |
| GADD45A | growth arrest & DNA damage induc. alpha | **3.12** | 1.79 | -2.48 | -3.64 | -4.84 |
| MLLT1 | MLLT1 super elongation complex SU | **2.76** | -1.14 | -1.89 | -1.7 | -2.68 |
| MIR646HG | MIR646 host gene | **2.55** | 1.19 | -1.29 | -2.24 | -3.96 |
| AGFG1 | ArfGAP with FG repeats 1 | **2.52** | 1.32 | -1.84 | -2.14 | -2.62 |
| KREMEN1 | kringle cont. transmembrane protein 1 | **2.52** | 1.44 | -3.02 | -1.37 | -12.89 |
| RIOK3 | RIO kinase 3 | -1.28 | **3.65** | -1.08 | -1.92 | -2.67 |
| BNIP3L | BCL2 interacting protein 3 like | -1.16 | **3.76** | -1.21 | -1.85 | -4.49 |
| TLCD4 | TLC domain containing 4 | -1.57 | **5.53** | -1.34 | -2.1 | -6.21 |
| SPTA1 | Spectrin alpha, erythrocytic 1 | -1.45 | **11.23** | -1.24 | -2.54 | -4 |
| TSPAN5 | tetraspanin 5 | -1.34 | **3.93** | -1.06 | -1.99 | -3.55 |
| GLRX5 | glutaredoxin 5 | -1.55 | **4.87** | -1.23 | -2.18 | -3.25 |
| IFIT1B | Interferon induced protein with tetratricopeptide repeats 1B | -2.27 | **7.29** | -1.47 | -2.26 | -6.05 |
| ADAM23 | ADAM metallopeptidase domain 23 | -1.94 | -2.85 | **2.81** | -1.06 | 1.26 |
| MAP7 | microtubule associated protein 7 | 1.08 | -1.51 | **2.28** | -1.42 | -2.02 |
| CACNA2D3 | Ca voltage-gated channel aux. Subunit Alpha2Delta3 | 1.22 | -2.05 | **1.91** | -1.07 | -2.45 |
| GPR34 | G protein-coupled receptor 34 | 1.02 | -1.36 | **2.42** | -1.33 | -2.88 |
| GRAMD1C | GRAM domain containing 1C | 1.27 | -1.44 | **2.69** | -2.15 | -2.6 |
| PLCB1 | phospholipase C Beta 1 | -1.1 | -1.41 | **1.8** | -1.06 | -1.36 |
| DYNC2H1 | dynein cytoplasmic 2 heavy chain 1 | -1.36 | -1.18 | **1.79** | -1.18 | 1.11 |
| TPRG1 | tumor protein p63 regulated 1 | -1.69 | -1.5 | **1.8** | -1 | 1.46 |
| ZNF600 | zinc finger protein 600 | -1.67 | -1.21 | **1.64** | 1.02 | 1.41 |
| PLEKHO1 | pleckstrin homology domain cont. O1 | -1.2 | -1.17 | -1.01 | **1.58** | -1.6 |
| APOL1 | apolipoprotein L1 | -1.23 | 1.58 | -2.61 | **1.68** | -1.59 |
| EPSTI1 | epithelial stromal interaction 1 | -1.64 | 1.72 | -3.56 | **1.99** | -1.26 |
| RSAD2 | radical S-adenosyl methionine domain containing 2 | -3.6 | 2.26 | -6.26 | **2.86** | -1.57 |
| IFITM3 | interferon induced transmembrane prot. 3 | 1.09 | 1.37 | -3.84 | **1.71** | -2.19 |
| SERPING1 | serpin family G member 1 | -1.83 | 1.93 | -7.13 | **2.38** | -1.59 |
| TPPP3 | tubulin polym. promoting protein FM3 | -1.12 | -2.35 | 1.41 | **1.67** | -2.37 |
| GTSE1 | G2 and S-phase expressed 1 | -3.7 | 1.4 | -2.37 | -1.3 | **6.49** |
| CDC45 | cell division cycle 45 | -3.88 | -1.05 | -1.97 | -1.21 | **6.86** |
| CENPF | centromere protein F | -2.86 | 1.73 | -2.04 | -1.45 | **5.12** |
| KIF14 | kinesin family member 14 | -2.88 | 1.5 | -2.23 | -1.54 | **5.93** |
| PDIA4 | protein disulfide isomerase FM A4 | -1.61 | -1.74 | -1.45 | -1.06 | **4.37** |
| KIF15 | kinesin family member 15 | -3.72 | 1.54 | -1.92 | -1.52 | **6.31** |

**Table S8a. Top performing gene pairs predicting each endotype in the ER discovery cohort.** Gene pairs were selected from the 40-gene multinomial classifier. The top five gene pairs that predicted each endotype are presented.

| **Endotype Prediction** | **Gene Pair** | **AUC ≡ Accuracy (%)** | **Sensitivity (%)** | **Specificity (%)** |
| --- | --- | --- | --- | --- |
| NPS vs Rest | MLLT1/MIR646HG | 96.9 | 94.2 | 88.2 |
|  | MLLT1/NSUN7 | 96.5 | 93 | 91.2 |
|  | IL1R1/MLLT1 | 96.4 | 94.2 | 87.2 |
|  | ATP9A/MLLT1 | 96.1 | 90.1 | 90.4 |
|  | GADD45A/MLLT1 | 96.1 | 89.7 | 90.9 |
| INF vs Rest | RIOK3/IFIT1B | 95.3 | 85.3 | 87.3 |
|  | SPTA1/GLRX5 | 95.3 | 90 | 85.1 |
|  | GLRX5/IFIT1B | 95.1 | 90.3 | 85.2 |
|  | SPTA1/IFIT1B | 94.7 | 90.8 | 86.7 |
|  | TLCD4/GLRX5 | 94.5 | 89.2 | 84.7 |
| IHD vs Rest | MAP7/PLCB1 | 90.3 | 85.2 | 79.2 |
|  | GRAMD1C/TPRG1 | 86.8 | 81.8 | 80.4 |
|  | ADAM23/MAP7 | 88.3 | 83.5 | 79.1 |
|  | GRAMD1C/PLCB1 | 88.7 | 78 | 80 |
|  | PLCB1/DYNC2H1 | 88.7 | 81.7 | 78.4 |
| IFN vs Rest | PLEKHO1/EPSTI1 | 85.8 | 82.6 | 75.8 |
|  | PLEKHO1/RSAD2 | 85.5 | 81.4 | 75 |
|  | PLEKHO1/SERPING1 | 84.4 | 78.8 | 74.4 |
|  | PLEKHO1/APOL1 | 82.9 | 73.5 | 73.4 |
|  | EPSTI1/TPPP3 | 81.6 | 78.5 | 70.1 |
| ADA vs Rest | GTSE1/PDIA4 | 97.1 | 86.7 | 89.2 |
|  | PDIA4/KIF15 | 97 | 83.9 | 91.2 |
|  | CENPF/PDIA4 | 96.9 | 86.9 | 90.7 |
|  | KIF14/PDIA4 | 96.8 | 86.5 | 89.6 |
|  | CENPF/KIF14 | 95 | 87.3 | 86.2 |

**Table S8b. Gene pair performance predicting each endotype in the ER discovery cohort.** Gene pairs were selected from the 40-gene multinomial classifier. In total 148 gene pairs from the 40-gene multinomial classifier were assessed. The remaining 123 gene pairs enabling prediction of each endotype with >75% accuracy are presented.

| **Comparison** | **Gene Set** | **AUC ≡ Accuracy (%)** | **Sensitivity (%)** | **Specificity (%)** |
| --- | --- | --- | --- | --- |
| **NPS-specific Gene Pairs** | | | | |
| NPS vs. Rest | PFKFB2/MLLT1 | 96 | 89.3 | 89.9 |
| NPS vs. Rest | MLLT1/ANXA3 | 96 | 91.3 | 87.9 |
| NPS vs. Rest | MLLT1/KREMEN1 | 96 | 93.6 | 87.6 |
| NPS vs. Rest | ARG1/MLLT1 | 95.9 | 87.9 | 89.6 |
| NPS vs. Rest | MLLT1/AGFG1 | 95.6 | 90.3 | 89.6 |
| NPS vs. Rest | NSUN7/KREMEN1 | 93.9 | 91.8 | 87.8 |
| NPS vs. Rest | NSUN7/MIR646HG | 93.8 | 87.1 | 85.6 |
| NPS vs. Rest | ANXA3/NSUN7 | 93.6 | 91.3 | 83.8 |
| NPS vs. Rest | ATP9A/NSUN7 | 93.4 | 92.6 | 87.1 |
| NPS vs. Rest | IL1R1/NSUN7 | 93.4 | 89.4 | 84.9 |
| NPS vs. Rest | AGFG1/NSUN7 | 93.4 | 86.9 | 87.2 |
| NPS vs. Rest | PFKFB2/NSUN7 | 93.1 | 88.8 | 86.2 |
| NPS vs. Rest | GADD45A/NSUN7 | 93 | 88.8 | 86.4 |
| NPS vs. Rest | ARG1/NSUN7 | 93 | 88.9 | 84.6 |
| NPS vs. Rest | ATP9A/KREMEN1 | 92.5 | 88.4 | 81.9 |
| NPS vs. Rest | ATP9A/IL1R1 | 92.4 | 92.8 | 84 |
| NPS vs. Rest | KREMEN1/MIR646HG | 92.2 | 87.8 | 83.9 |
| NPS vs. Rest | ATP9A/MIR646HG | 92 | 86.2 | 84.4 |
| NPS vs. Rest | IL1R1/ANXA3 | 91.9 | 90.4 | 80.6 |
| NPS vs. Rest | ANXA3/MIR646HG | 91.8 | 87 | 81.1 |
| NPS vs. Rest | IL1R1/AGFG1 | 91.7 | 86.6 | 81 |
| NPS vs. Rest | AGFG1/KREMEN1 | 91.5 | 87.8 | 81.6 |
| NPS vs. Rest | ATP9A/ANXA3 | 91.3 | 85.5 | 81 |
| NPS vs. Rest | IL1R1/KREMEN1 | 91.2 | 88.6 | 82 |
| NPS vs. Rest | GADD45A/KREMEN1 | 91 | 87.9 | 81.3 |
| NPS vs. Rest | ATP9A/AGFG1 | 90.9 | 82.2 | 82.9 |
| NPS vs. Rest | IL1R1/MIR646HG | 90.7 | 83.4 | 81.1 |
| NPS vs. Rest | ATP9A/GADD45A | 90.5 | 82.5 | 82.3 |
| NPS vs. Rest | ATP9A/PFKFB2 | 90.5 | 80.3 | 82.5 |
| NPS vs. Rest | IL1R1/GADD45A | 90.5 | 86.6 | 81.8 |
| NPS vs. Rest | ANXA3/AGFG1 | 90.4 | 86.6 | 80.1 |
| NPS vs. Rest | ANXA3/KREMEN1 | 90.4 | 90.9 | 76.4 |
| NPS vs. Rest | AGFG1/MIR646HG | 90.3 | 79.4 | 78.3 |
| NPS vs. Rest | ATP9A/ARG1 | 89.7 | 82.3 | 81.7 |
| NPS vs. Rest | GADD45A/ANXA3 | 89.6 | 85.7 | 79.3 |
| NPS vs. Rest | PFKFB2/KREMEN1 | 89.6 | 84.5 | 79.4 |
| NPS vs. Rest | IL1R1/PFKFB2 | 89.5 | 87.7 | 80 |
| NPS vs. Rest | GADD45A/MIR646HG | 89.1 | 78.4 | 78.9 |
| NPS vs. Rest | GADD45A/AGFG1 | 88.8 | 81.2 | 79.8 |
| NPS vs. Rest | PFKFB2/ANXA3 | 88.8 | 86.2 | 78 |
| NPS vs. Rest | IL1R1/ARG1 | 88.7 | 82.8 | 78.4 |
| NPS vs. Rest | ARG1/KREMEN1 | 88.7 | 83.1 | 77.1 |
| NPS vs. Rest | ARG1/ANXA3 | 88.2 | 87.8 | 78.5 |
| NPS vs. Rest | PFKFB2/AGFG1 | 88.2 | 79.6 | 79.1 |
| NPS vs. Rest | PFKFB2/MIR646HG | 88.2 | 78.8 | 78.5 |
| NPS vs. Rest | ARG1/AGFG1 | 87.3 | 82.9 | 79.6 |
| NPS vs. Rest | GADD45A/PFKFB2 | 86.8 | 78.6 | 81.2 |
| NPS vs. Rest | ARG1/MIR646HG | 85.4 | 75.6 | 76.1 |
| NPS vs. Rest | GADD45A/ARG1 | 85.3 | 76.2 | 79.7 |
| NPS vs. Rest | ARG1/PFKFB2 | 83.5 | 76.8 | 75.8 |
| **INF-specific Gene Pairs** | | | | |
| INF vs. Rest | RIOK3/IFIT1B | 95.3 | 85.3 | 87.3 |
| INF vs. Rest | SPTA1/GLRX5 | 95.3 | 90 | 85.1 |
| INF vs. Rest | GLRX5/IFIT1B | 95.1 | 90.3 | 85.2 |
| INF vs. Rest | RIOK3/GLRX5 | 94.8 | 85.4 | 85.6 |
| INF vs. Rest | TLCD4/IFIT1B | 94.8 | 89.7 | 84 |
| INF vs. Rest | SPTA1/IFIT1B | 94.7 | 90.8 | 86.7 |
| INF vs. Rest | RIOK3/SPTA1 | 94.5 | 88.2 | 86.1 |
| INF vs. Rest | TLCD4/GLRX5 | 94.5 | 89.2 | 84.7 |
| INF vs. Rest | BNIP3L/IFIT1B | 94.4 | 91.2 | 85.6 |
| INF vs. Rest | BNIP3L/GLRX5 | 94.3 | 87.8 | 84.6 |
| INF vs. Rest | BNIP3L/SPTA1 | 94.1 | 86.5 | 84.1 |
| INF vs. Rest | RIOK3/TLCD4 | 93.8 | 87 | 84 |
| INF vs. Rest | TLCD4/SPTA1 | 93.8 | 86.6 | 82.1 |
| INF vs. Rest | RIOK3/BNIP3L | 93.7 | 86.8 | 85 |
| INF vs. Rest | TSPAN5/GLRX5 | 93.6 | 87.2 | 84.8 |
| INF vs. Rest | TSPAN5/IFIT1B | 93.6 | 83.1 | 84.6 |
| INF vs. Rest | BNIP3L/TLCD4 | 93.2 | 86.2 | 81.5 |
| INF vs. Rest | SPTA1/TSPAN5 | 93 | 83.7 | 84.2 |
| INF vs. Rest | RIOK3/TSPAN5 | 92.8 | 82.8 | 84.3 |
| INF vs. Rest | TLCD4/TSPAN5 | 92.2 | 83.7 | 82.7 |
| INF vs. Rest | BNIP3L/TSPAN5 | 92.1 | 82.2 | 82.5 |
| **IHD-specific Gene Pairs** | | | | |
| IHD vs. Rest | MAP7/PLCB1 | 90.3 | 85.2 | 79.2 |
| IHD vs. Rest | GPR34/PLCB1 | 89.2 | 88.3 | 76.6 |
| IHD vs. Rest | PLCB1/TPRG1 | 89.1 | 83.2 | 76.1 |
| IHD vs. Rest | GRAMD1C/PLCB1 | 88.7 | 78 | 80 |
| IHD vs. Rest | PLCB1/DYNC2H1 | 88.7 | 81.7 | 78.4 |
| IHD vs. Rest | ADAM23/MAP7 | 88.3 | 83.5 | 79.1 |
| IHD vs. Rest | GPR34/TPRG1 | 87.6 | 84.3 | 76.2 |
| IHD vs. Rest | PLCB1/ZNF600 | 87.4 | 84.8 | 76.2 |
| IHD vs. Rest | ADAM23/GRAMD1C | 87.3 | 81.5 | 79.2 |
| IHD vs. Rest | ADAM23/PLCB1 | 86.9 | 83 | 74.4 |
| IHD vs. Rest | GRAMD1C/TPRG1 | 86.8 | 81.8 | 80.4 |
| IHD vs. Rest | MAP7/DYNC2H1 | 86.6 | 74.2 | 77.6 |
| IHD vs. Rest | CACNA2D3/PLCB1 | 86.5 | 88.7 | 72.7 |
| IHD vs. Rest | MAP7/ZNF600 | 85.9 | 77.9 | 76.8 |
| IHD vs. Rest | ADAM23/GPR34 | 85.7 | 79.5 | 74.4 |
| IHD vs. Rest | CACNA2D3/TPRG1 | 85.5 | 82.4 | 72.9 |
| IHD vs. Rest | MAP7/GPR34 | 85.2 | 78.5 | 74.6 |
| IHD vs. Rest | MAP7/TPRG1 | 84.4 | 70.8 | 77.8 |
| IHD vs. Rest | GRAMD1C/ZNF600 | 84.2 | 77 | 77.1 |
| IHD vs. Rest | ADAM23/CACNA2D3 | 84.1 | 84.2 | 72.4 |
| IHD vs. Rest | GPR34/GRAMD1C | 84 | 73.8 | 74 |
| IHD vs. Rest | MAP7/GRAMD1C | 83.8 | 71.8 | 79.6 |
| IHD vs. Rest | CACNA2D3/GRAMD1C | 83.8 | 78.4 | 75.8 |
| IHD vs. Rest | GPR34/DYNC2H1 | 83.8 | 69.8 | 79.1 |
| IHD vs. Rest | CACNA2D3/ZNF600 | 83.7 | 81.5 | 73.3 |
| IHD vs. Rest | GPR34/ZNF600 | 83.7 | 73.6 | 76.6 |
| IHD vs. Rest | CACNA2D3/DYNC2H1 | 83.4 | 74.1 | 75.4 |
| IHD vs. Rest | GRAMD1C/DYNC2H1 | 83.2 | 71.8 | 78.7 |
| IHD vs. Rest | MAP7/CACNA2D3 | 82.7 | 73.8 | 73.5 |
| IHD vs. Rest | ADAM23/DYNC2H1 | 82.5 | 68.4 | 76.1 |
| IHD vs. Rest | CACNA2D3/GPR34 | 82.4 | 80.9 | 71.4 |
| IHD vs. Rest | DYNC2H1/TPRG1 | 82.3 | 69.7 | 75.8 |
| IHD vs. Rest | ADAM23/TPRG1 | 81.6 | 72.9 | 70.6 |
| IHD vs. Rest | DYNC2H1/ZNF600 | 81.5 | 72.8 | 77.3 |
| IHD vs. Rest | ADAM23/ZNF600 | 80.8 | 69.6 | 73.7 |
| IHD vs. Rest | TPRG1/ZNF600 | 79.4 | 73.1 | 68.5 |
| **IFN-specific Gene pairs** | | | | |
| IFN vs. Rest | PLEKHO1/EPSTI1 | 85.8 | 82.6 | 75.8 |
| IFN vs. Rest | PLEKHO1/RSAD2 | 85.5 | 81.4 | 75 |
| IFN vs. Rest | PLEKHO1/SERPING1 | 84.4 | 78.8 | 74.4 |
| IFN vs. Rest | PLEKHO1/APOL1 | 82.9 | 73.5 | 73.4 |
| IFN vs. Rest | EPSTI1/TPPP3 | 81.6 | 78.5 | 70.1 |
| IFN vs. Rest | SERPING1/TPPP3 | 81.6 | 75.1 | 72.6 |
| IFN vs. Rest | PLEKHO1/IFITM3 | 80.6 | 78.8 | 70.7 |
| IFN vs. Rest | RSAD2/TPPP3 | 80.5 | 71.2 | 71.6 |
| IFN vs. Rest | APOL1/TPPP3 | 79.9 | 68.3 | 72.3 |
| IFN vs. Rest | RSAD2/SERPING1 | 79.9 | 78.9 | 71.1 |
| IFN vs. Rest | PLEKHO1/TPPP3 | 79.7 | 78 | 71 |
| IFN vs. Rest | APOL1/SERPING1 | 79.6 | 76.8 | 71.5 |
| IFN vs. Rest | EPSTI1/SERPING1 | 79.5 | 79.5 | 70.2 |
| IFN vs. Rest | APOL1/EPSTI1 | 79.1 | 76.4 | 70.2 |
| IFN vs. Rest | APOL1/RSAD2 | 79.1 | 77 | 73.2 |
| IFN vs. Rest | IFITM3/SERPING1 | 78.8 | 75.2 | 72.4 |
| IFN vs. Rest | EPSTI1/RSAD2 | 78.2 | 79.9 | 68.2 |
| IFN vs. Rest | EPSTI1/IFITM3 | 77.3 | 80.5 | 68.3 |
| IFN vs. Rest | APOL1/IFITM3 | 77.2 | 67.6 | 71.5 |
| IFN vs. Rest | RSAD2/IFITM3 | 76.5 | 70.2 | 70.1 |
| IFN vs. Rest | IFITM3/TPPP3 | 70.6 | 65.6 | 62.8 |
| **ADA-specific Gene Pairs** | | | | |
| ADA vs. Rest | GTSE1/PDIA4 | 97.1 | 86.7 | 89.2 |
| ADA vs. Rest | PDIA4/KIF15 | 97 | 83.9 | 91.2 |
| ADA vs. Rest | CENPF/PDIA4 | 96.9 | 86.9 | 90.7 |
| ADA vs. Rest | KIF14/PDIA4 | 96.8 | 86.5 | 89.6 |
| ADA vs. Rest | CDC45/PDIA4 | 96.6 | 83.2 | 91.1 |
| ADA vs. Rest | GTSE1/KIF14 | 96.4 | 84.5 | 90.1 |
| ADA vs. Rest | GTSE1/CDC45 | 96.3 | 84.8 | 89 |
| ADA vs. Rest | GTSE1/KIF15 | 96.2 | 85.5 | 88.8 |
| ADA vs. Rest | GTSE1/CENPF | 96.1 | 84.6 | 89.3 |
| ADA vs. Rest | CDC45/KIF14 | 95.8 | 84.5 | 89.2 |
| ADA vs. Rest | CDC45/CENPF | 95.6 | 81.8 | 87.6 |
| ADA vs. Rest | CDC45/KIF15 | 95.2 | 82.4 | 87.9 |
| ADA vs. Rest | CENPF/KIF15 | 95.2 | 82.3 | 86.3 |
| ADA vs. Rest | CENPF/KIF14 | 95 | 87.3 | 86.2 |
| ADA vs. Rest | KIF14/KIF15 | 94.7 | 84.3 | 85.5 |

**Table S9. Severity and outcomes of the endotypes in the ER validation cohort.** The mean value, standard error, and total available observations for numerical variables, i.e. mean ± standard error (n). Categorical variables are presented as percent positive (positive/n), i.e. percent (total positive/total available observations). ER = value in the Emergency Department at first clinical presentation.

| **Parameter** | **Mechanistic Endotypes** | | | | |  |
| --- | --- | --- | --- | --- | --- | --- |
|  | **NPS (N=25)** | **INF (N=18)** | **IHD (N=19)** | **IFN (N=19)** | **ADA (N=3)** | **P value** |
| ER Altered Mental State | 32% (8/25) | 0% (0/18) | 5·3% (1/19) | 0% (0/19) | 0% (0/3) | 0·0060 |
| ER Lactate (mmol/L) | 1·7 ± 0·28 (23) | 2·2 ± 0·45 (16) | 1·1 ± 0·11 (14) | 0·9 ± 0·1 (12) | 1 ± 0·05 (2) | 0·018 |
| ER Aspartate aminotransferase (AST) (U/L) | 70·7 ± 24·24 (23) | 47·1 ± 9·37 (16) | 55·4 ± 19·08 (14) | 23·8 ± 2·91 (18) | 74·7 ± 53·17 (3) | 0·035 |
| ER SOFA Score | 2·8 ± 0·49 (25) | 3·1 ± 0·57 (18) | 1·8 ± 0·45 (19) | 1·2 ± 0·29 (19) | 2·3 ± 1·45 (3) | 0·056 |
| Age (years) | 62·5 ± 3·84 (25) | 57·6 ± 3·82 (18) | 61·5 ± 4·26 (19) | 46·8 ± 5·07 (19) | 42·7 ± 12·68 (3) | 0·056 |
| ER Alkaline phosphatase (ALP) (U/L) | 115·4 ± 17·06 (23) | 143·2 ± 28·05 (16) | 104·8 ± 11·99 (14) | 91·2 ± 15·68 (18) | 88·7 ± 19·13 (3) | 0·061 |
| ER Urea (mmol/L) | 9·8 ± 1·35 (24) | 6 ± 0·96 (18) | 6·6 ± 1·01 (16) | 4·9 ± 0·5 (18) | 8·9 ± 4·87 (3) | 0·067 |
| ER Bands Greater than 5% | 20% (5/25) | 50% (9/18) | 23·5% (4/17) | 16·7% (3/18) | 66·7% (2/3) | 0·07 |
| Hospital Stay Days | 9·7 ± 1·88 (25) | 4·8 ± 0·79 (18) | 5·9 ± 1·39 (19) | 4·4 ± 1·2 (19) | 2 ± 1·15 (3) | 0·072 |
| ER MAP  (mmHg) | 76·6 ± 2·15 (25) | 81·5 ± 2·73 (18) | 89·7 ± 4·02 (19) | 82·5 ± 3·36 (18) | 86·6 ± 3·26 (3) | 0·075 |
| ER Temperature (Celcius) | 38·4 ± 0·19 (24) | 38·7 ± 0·25 (18) | 38 ± 0·17 (18) | 38·7 ± 0·21 (19) | 38·1 ± 0·55 (3) | 0·095 |
| ER Billirubin (mg/dl) | 16·6 ± 2·17 (24) | 32·7 ± 17·08 (17) | 10·8 ± 2·16 (16) | 14·4 ± 2·5 (18) | 8 ± 2·52 (3) | 0·11 |
| Duration of Illness before ER Arrival | 3·6 ± 1·21 (25) | 4·4 ± 2·38 (18) | 3·1 ± 0·95 (19) | 4·7 ± 1·27 (18) | 5 ± 1·53 (3) | 0·11 |
| ER Heart Rate (beats/min) | 110·4 ± 4·59 (25) | 118·7 ± 2·98 (18) | 101 ± 4·76 (19) | 113·6 ± 5·32 (18) | 103·3 ± 17·64 (3) | 0·11 |
| Mortality | 8% (2/25) | 27·8% (5/18) | 5·3% (1/19) | 5·3% (1/19) | 0% (0/3) | 0·11 |
| ER Creatinine (mg/dl) | 118 ± 11·1 (25) | 94·8 ± 15·71 (18) | 92·4 ± 10·2 (19) | 83·9 ± 3·95 (18) | 182·7 ± 91·85 (3) | 0·15 |
| Treatment – Oxygen Therapy | 36% (9/25) | 16·7% (3/18) | 47·4% (9/19) | 27·8% (5/18) | 0% (0/3) | 0·20 |
| Sex (% female) | 40% (10/25) | 61·1% (11/18) | 57·9% (11/19) | 57·9% (11/19) | 0% (0/3) | 0·21 |
| ER Alanine Aminotransferase (IU/L) | 56·1 ± 13·58 (23) | 46·6 ± 8·17 (16) | 50·4 ± 11·07 (14) | 27·4 ± 3·59 (18) | 97·7 ± 66·35 (3) | 0·21 |
| ER qSOFA | 1·1 ± 0·2 (25) | 0·7 ± 0·14 (18) | 0·6 ± 0·17 (19) | 0·5 ± 0·14 (19) | 0·3 ± 0·33 (3) | 0·23 |
| ER Respiratory Rate (breaths/min) | 22·5 ± 1·13 (24) | 21·4 ± 1·33 (18) | 21·6 ± 1·37 (19) | 19 ± 0·7 (19) | 19 ± 3·51 (3) | 0·23 |
| ER Diastolic (mmHg) | 65·4 ± 2·37 (23) | 67·3 ± 2·75 (18) | 67·3 ± 3·7 (19) | 70·9 ± 2·76 (18) | 76·7 ± 2·33 (3) | 0·31 |
| On Antibiotics before ER Arrival | 20% (5/25) | 33·3% (6/18) | 42·1% (8/19) | 26·3% (5/19) | 0% (0/3) | 0·38 |
| Within 72 SOFA | 2·4 ± 0·59 (25) | 2·1 ± 0·62 (18) | 1·4 ± 0·41 (19) | 1·2 ± 0·48 (19) | 1 ± 0·58 (3) | 0·43 |
| ER Systolic (mmHg) | 110·4 ± 4·22 (25) | 117·1 ± 5·5 (18) | 124·1 ± 5·44 (19) | 117·2 ± 5·92 (18) | 117·3 ± 6·06 (3) | 0·48 |
| Blood Culture Result | 24% (6/25) | 29·4% (5/17) | 16·7% (3/18) | 11·1% (2/18) | 0% (0/3) | 0·57 |
| ER HCO3 (mmol/L) | 22·6 ± 0·85 (21) | 24·2 ± 1·1 (15) | 24·6 ± 1·23 (16) | 23·9 ± 0·7 (17) | 23·1 ± 0·05 (2) | 0·64 |
| Treatment – Antibiotics | 88% (22/25) | 83·3% (15/18) | 89·5% (17/19) | 78·9% (15/19) | 66·7% (2/3) | 0·72 |
| ER FIO_2_ (%) | 29 ± 3·73 (21) | 29 ± 5·07 (16) | 27·2 ± 3·96 (17) | 26 ± 4·56 (16) | 21 ± 0 (2) | 0·83 |
| Readmit Within 28 Days | 20% (5/25) | 11·1% (2/18) | 10·5% (2/19) | 15·8% (3/19) | 0% (0/3) | 0·87 |

**Table S10. Severity and outcomes of the endotypes in the ICU validation cohort.** The mean value, standard error, and total available observations for numerical variables, i.e. mean ± standard error (n). Categorical variables are presented as percent positive (positive/n), i.e. percent (total positive/total available observations). P values derived from Kruskal-Wallis and Chi square tests testing for significant differences between endotypes.

| **Parameter** | **Mechanistic Endotypes** | | | | **P Value** |
| --- | --- | --- | --- | --- | --- |
|  | **NPS (N=36)** | **INF (N=33)** | **IHD (N=6)** | **IFN (N=7)** |  |
| COVID-19 PCR Positivity | 16·7% (6/36) | 39·4% (13/33) | 16·7% (1/6) | 100% (7/7) | 0·00050 |
| Mortality within 28 Days | 45·7% (16/35) | 25·9% (7/27) | 0% (0/5) | 0% (0/6) | 0·025 |
| SOFA 24H post ICU admission | 7·6 ± 0·9 (34) | 8·2 ± 0·78 (32) | 3·5 ± 1·34 (6) | 3·7 ± 1·49 (7) | 0·033 |
| ICU Mortality | 38·9% (14/36) | 18·2% (6/33) | 0% (0/6) | 0% (0/5) | 0·034 |
| ICU Stay Days | 10·4 ± 1·29 (36) | 15·2 ± 1·63 (33) | 6·8 ± 2·7 (6) | 9·7 ± 3·43 (7) | 0·050 |
| SOFA 48H post admission | 7·5 ± 0·98 (31) | 8·4 ± 0·75 (30) | 3·5 ± 0·87 (4) | 4·1 ± 1·7 (7) | 0·079 |
| SOFA at ICU admission | 8·4 ± 0·9 (36) | 7·9 ± 0·64 (33) | 4·2 ± 1·7 (6) | 5 ± 1·66 (7) | 0·093 |
| Treatment – Antibiotics | 77·8% (28/36) | 87·9% (29/33) | 50% (3/6) | 71·4% (5/7) | 0·13 |
| Duration of Illness before ICU Arrival (days) | 5·6 ± 1·56 (24) | 7·9 ± 1·46 (25) | 2·7 ± 0·95 (6) | 4·1 ± 1·14 (7) | 0·10 |
| Age (years) | 59·6 ± 2·41 (36) | 62·8 ± 2·81 (33) | 70·2 ± 5·83 (6) | 59·9 ± 6·78 (7) | 0·47 |
| Blood Culture Result | 16·7% (6/36) | 9·1% (3/33) | 0% (0/6) | 14·3% (1/7) | 0·69 |
| Sex (% Female) | 25% (9/36) | 36·4% (12/33) | 33·3% (2/6) | 28·6% (2/7) | 0·81 |

***Supplemental Methods***

*RNA-Seq processing*

A standard RNA-Seq processing protocol was used, including quality control using fastqc (v0.11.7)^45^ and multiqc (v1.6)^46,^ alignment to the human genome (Ensembl GRCh38.92) using STAR (v2.6.0a),^47^ and read count assessments using htseq-count (v0.11.0).^48^ Finally, globin genes and genes with fewer than 10 counts were removed from count tables, and samples with fewer than one million total counts were not further analyzed. A variance stabilizing transformation (VST) was performed to render counts homoscedastic and normalized for varying library sizes.^18^ Following transformation, technical variation due to sequencing batch was removed using ComBat within the Surrogate Variable Analysis R package (3.30.1).^49^ Gene expression from the discovery and validation cohorts were treated independently of each other prior to VST normalization and batch correction to avoid signal leakage (also referred to as train-test leakage).

***Supplemental Results***

*Discovery and validation groups identified five clusters with distinct biological mechanisms and clinical outcomes*

Based on the resultant cluster validation metrics, it was determined that patients could be stratified into one of five clusters, which could best compartmentalize the various sources of heterogeneity observed in sepsis patients. Based on the training data set of 182 patients from Netherlands, Colombia and Vancouver, Canada, Clusters one to five were comprised of 29, 58, 26, 48, and 21 patients, respectively. These clusters were considered to be endotypes, which are subtypes of a complex condition, in this case sepsis, that are defined by distinct functional mechanisms and clinical outcomes. The dominant biological mechanisms of each endotype were initially characterized by comparison to a set of healthy controls (n = 27). Specifically differential expression was performed, followed by overrepresentation analysis using Reactome^19,50^ and MSigDB^20^ biological programs. Endotypes were named Neutrophilic-Suppressive (NPS), Inflammatory (INF), Innate Host Defence (IHD), Interferon (IFN), and Adaptive (ADA) based on pathway analysis. Clinical measurements were compared between clusters using ranked analysis of variance statistics ( Kruskal-Wallis tests) and Chi-square tests depending on the variable type. Variables with p values ≤0·05 were considered significant. In particular, the clusters were compared in the context of impending severity and outcomes, as measured by SOFA scores measured 24 and 72 hours post admission, length of hospital stay, treatments, ICU admission and mortality (Table S5). Each cluster had unique mechanistic underpinnings and varied in symptomology and outcomes, indicative of clinically-relevant endotypes.

Subsequently gene expression signatures were identified by comparing global gene expression profiles between endotypes using differential expression analysis (top 200 differentially expressed genes when comparing each endotype to all others). This reflected the unique biological character of each endotype as revealed by plotting the gene expression differences onto protein:protein interaction (PPI) networks using NetworkAnalyst^44^ (Figure S4). Since PPI reveal the functionally-based interactions of individual proteins, the formation of tight and discrete networks indicates strong mechanistic differences between individual endotypes. In addition, gene set variation analysis (GSVA, an unsupervised method that calculates per sample enrichment scores as a function of gene expression inside and outside the gene set)^30^ was used to assess the enrichment of the ~200 gene unique signatures in each patient, which demonstrated that a signature corresponding to a single associated endotype was highly enriched in each of the classified patients (Table S6). A classification scheme was derived for the endotype model using a supervised machine learning model, namely multinomial regression with LASSO regularization (AUC/accuracy: 96%; Sensitivity: 81%; Specificity: 95%). LASSO was used to shrink particular coefficient estimates to zero, yielding a simple/sparse model with a minimized set of gene expression events discriminating each endotype from all others. The trained multinomial endotype model was applied to each patient’s gene expression profile (using the genes and model parameters) in the ER and ICU validation cohorts to predict endotype status. There were 40 genes selected, representing a minimal signature to classify patients into endotypes (Table S7). The reduced 40 gene signature showed clear expression patterns with respect to the endotypes, indicating the minimal set of genes accurately differentiated each endotype (Figure S3).

To further facilitate translation to clinics, we assessed whether much smaller pairs of genes from the 40-gene multinomial model could stratify patients into endotypes from the discovery cohort. Specifically, the genes differentially expressed in each endotype were used to predict a specific endotype compared to all others (i.e. 11 DE NPS genes = 55 gene pairs assessed). In total, 148 gene pairs were assessed for predictive performance using logistic regression. The top five gene pairs for each endotype comparison are presented in Table S8a with the remaining gene pairs in Table 8b, and represent additional markers for accurate endotype prediction.

The validation cohorts were used to determine if the unique endotype characteristics and clinical trends observed in the discovery set were stable, thereby providing support for the generalizability and clinical usefulness of the endotype model. The model was first validated by application to a validation cohort of 84 patients from Sydney, Australia. For this cohort sample collection had followed the same inclusion and exclusion criteria as the discovery group. The endotype classification model demonstrated that the patients could be separated into the same 5 endotypes (Figure S3; Table S9). More detailed analysis confirmed that the signatures captured similar biological associations, where we obtained dominant biological mechanisms of each endotype by comparison to a set of healthy controls (n = 12). Clinical associations were also similar, with the NPS and INF endotypes displaying worse outcomes. This encouraged us to then study all 266 patients (the ER cohort) to identify mechanistic patterns revealing the diverse immunological processes involved in each endotype (Figure S5; see also main manuscript).

We also applied the endotype classifier to a Toronto, Canada cohort of patients recruited from the ICU (with the exception of two patients recruited from the wards). This cohort demonstrated higher severity and poorer outcomes when compared to the ER cohort. Four of the five endotypes were evident, and the mechanistic and clinical trends were recapitulated (Table S10; see also main manuscript).

***Supplemental Discussion***

Our primary motivation was identifying endotypes and markers of severity in earliest stages of sepsis. We determined that there were baseline differences in expression of hundreds of genes between patients who progressed to more severe outcomes. These genes were related to neutrophil degranulation and various cytokine signaling pathways, but also platelet activity, cell surface interactions, and metabolism. Thus, severity associated genes represent a potentially useful way to predict, very early, sepsis outcomes in a binary fashion.

Endotypes offer an insight into the specific molecular dysregulation occurring, enabling specific prognostic markers and therapeutic options to prevent deterioration. Using consensus clustering we were able to test the hypothesis that robust mechanistically distinct clusters exist within suspected sepsis patients. Furthermore, to confirm that each cluster represents clinically relevant endotypes, we determined if the clusters were associated with clinical severity and outcomes. The endotype model stratified patients into one of five endotypes, each with a unique gene expression profile exhibiting diverse molecular responses and differential overall severity (see Main manuscript). Analysis of severity in the identified endotypes indicated that NPS and INF patients generally progressed to worse outcomes. However, this was not always the case when examining severity outcomes in individual patients, likely due to (successful) antibiotic or other treatments provided, nosocomial infections, and/or other factors resulting in rapid deterioration. The other three endotypes demonstrated distinct and novel mechanisms, and tended to cluster to some extent on principal component analysis while demonstrating significantly lower ER SOFA scores, and 11 other clinical parameters. Of these the ADA endotype was associated with substantially younger patients who showed down-regulation of the predictive CR signature, rapid resolution of SOFA scores, higher predicted levels of lymphocytes and upregulation of B-cell pathways, and was not identified in ICU patients. The IFN and ADA endotypes displayed the overall best prognoses, and less severe clinical symptomology (e.g. lower SOFA scores) and outcomes, cf. other endotypes. The IFN endotype was particularly marked by an elevated expression of interferon signaling pathways that might reflect a viral etiology^55-56^ and/or reflect strong inflammatory/anti-viral responses.

We further validated our endotype model in ICU patients presenting with severe sepsis. Given the current pandemic, we had the unique opportunity to recruit ICU patients suspected of COVID-19, since severely-affected COVID-19 patients have been proposed to suffer from sepsis^.57-58^ Intriguingly the two most severe endotypes, NPS and INF, dominated the ICU with 69 out of 82 patients (84%) and were associated with significantly more severe outcomes (43% and 30% respectively). The ADA endotype was completely absent while another, the IFN endotype, was only found in 26% of COVID-19 patients and associated with lower SOFA scores, reduced hospital stay days and no mortality. The IHD endotype was found at a low frequency in both COVID-19 PCR positive (1) and negative (5) patients and like the IFN endotype reflected better outcomes and the shortest average ICU stay days.

Few studies have explored endotypes and associated clinical characteristics in adult sepsis to date and only with ICU patients.^13-15^ Maslove *et al* specifically profiled neutrophil gene expression, and indicated two endotypes, Subgroup1 and Subgroup 2.^13^ Subgroup 1 was associated with higher severity scores, and increased expression of key inflammation pathways in neutrophils, specifically, cytokine signaling pathways and Toll-like receptor (TLR) signaling. This study is generally consistent with our findings that showed an even earlier role for neutrophils albeit with additional complexity. Davenport *et al*^14^ identified the Sepsis Response Signature 1 (SRS1) and SRS2 endotypes, with SRS1 associated with higher mortality and immunosuppression, expressing endotoxin-tolerance/cellular reprogramming consistent with our published severity signature^20^ Similarly, Scicluna *et al*^15^ showed that their high-mortality Mars1 endotype displayed the hallmarks of immunosuppression, as did the NPS and INF endotypes in the ICU portion of the current study. Notably, the cellular underpinnings (in our case neutrophils) were different, and our study showed that endotypes were clearly present in the ER and showed a greater diversity in mechanisms.

We have already shown that the CR severity signature discriminates between sepsis patients and other ICU-bound patients but fails to identify seriously ill kidney and heart patients. Here we have extended this analysis and shown that the mechanisms underlying myocardial infarction (i.e. ST-elevated myocardial infarction/STEMI vs non-ST-elevated myocardial infarction/NSTEMI) and cancer/acute infection (i.e. Leukemia vs Lymphoma) patients are distinct from mechanisms captured by our severe sepsis signatures. Assignment with the CR signature (which showed the best performance in predicting severity) showed no association with myocardial infarction (93·3% Low and 6·7% High; Chi-square p= 1·0) or cancer (75·0% Low and 25·5% High; Chi-square p= 0·52). The endotype assignment of the myocardial infarction and cancer patients showed no association with the experimental groups of interest (described in Main manuscript). Accordingly, these analyses indicate that the mechanisms we identified are substantially specific for sepsis.

The signatures described in this study and those proposed by other groups were different, perhaps reflecting in part the different stages of sepsis from which signatures were derived (i.e. our study was performed on suspected sepsis patients in the ER while previous studies involved ICU patients with confirmed sepsis) as well as a lack of standardized methods used across research groups.^41^ Specifically, differences in the assay technologies used (microarrays vs. more accurate RNA-Seq) and the statistical/machine learning methods used for normalization, feature selection, and prediction can all, in part, influence the identification of different signatures. Nevertheless, although the specific genes within the signatures are clearly different, the pathways that they represent overlapped to some extent. With respect to the study we performed, we used the highly accurate method of RNA-Seq and included 5 cohorts from 4 continents, representing a substantial patient demographic. Furthermore, we characterized suspected sepsis/pre-sepsis patients who displayed a broader range of molecular responses, and collected extensive clinical data profiles. Accordingly, the signatures we propose are robust and applicable to a broad population, and in particular address patients at first clinical presentation (cf. the ICU in previous studies).

There are certain limitations in this study. The degree to which sepsis progresses over time depends on the complex interplay of the infection type/pathogen, therapeutic use and timing of antibiotics, immune-modulators and supportive therapies, and patient heterogeneity.^59-60^ Although we took samples as rapidly as possible after first clinical presentation, the study likely included patients in varying stages of sepsis or SIRS, and these symptoms typically appeared days prior to ER admission. It would be useful to track endotype status over time to see when they occur and if endotypes can interconvert. Interestingly, however, the duration of illness and antibiotic usage prior to ER admission were not significant in the endotypes over the discovery and validation cohorts. This was an international multi-center, prospective clinical trial analyzed by machine learning methods that consider location as one of many features. A large validation was used to confirm our classification methods but it is worth mentioning that the inhomogeneity of our cohorts should be taken into account. Future directions will involve measuring signature genes using reverse transcription polymerase chain reaction (RT-PCR), which better represents the technology used in clinical settings.

***Supplemental References***

45. Andrews S. FastQC: a quality control tool for high throughput sequence data.

46. Ewels P, Magnusson M, Lundin S, Käller M. MultiQC: summarize analysis results for multiple tools and samples in a single report. *Bioinformatic*. 2016;**32(19)**:3047–8.

47. Dobin A, Davis CA, Schlesinger F, et al. STAR: ultrafast universal RNA-seq aligner. Bioinformatics. 2012; 29(1):15–21. doi:10.1093/bioinformatics/bts635

48. Anders S, Pyl PT, Huber W. HTSeq-A Python framework to work with high-throughput sequencing data. Bioinformatics 2015; 31(2):166–69. doi:10.1093/bioinformatics/btu638

49. Leek JT, Johnson WE, Parker HS, Jaffe AE, Storey JD, Kelso J. The sva package for removing batch effects and other unwanted variation in high-throughput experiments. Bioinformatics Applications Note 2012;28(6): 882–883. doi:10.1093/bioinformatics/bts034

50. Yu G, He QY. ReactomePA: An R/Bioconductor package for reactome pathway analysis and visualization. Molecular BioSystems 2016;12(2):477–479. doi:10.1039/c5mb00663e

51. Tibshirani R, Walther G, Hastie T. Estimating the number of clusters in a data set via the gap statistic. J R Stat Soc 2001; 63(2):411–423. doi:10.1111/1467-9868.00293

52. Rousseeuw PJ. Silhouettes: A graphical aid to the interpretation and validation of cluster analysis. J Comput Appl Math 1987; 20(C): 53–65. doi:10.1016/0377-0427(87)90125-7

53. Handl J, Knowles J, Kell DB. Data and text mining Computational cluster validation in post-genomic data analysis. Bioinformatics Review 2005; 21(15):3201–3212. doi:10.1093/bioinformatics/bti517

54. Wilkerson MD, Hayes DN. ConsensusClusterPlus: a class discovery tool with confidence assessments and item tracking. Bioinformatics Applications Note 2010;26(12):1572–1573. doi:10.1093/bioinformatics/btq170

55. Li H, Liu L, Zhang D, et al. SARS-CoV-2 and viral sepsis: observations and hypotheses. *Lancet* 2020; 395: 1517–1520.

56. Lin GL, McGinley JP, Drysdale SB, Pollard AJ. Epidemiology and immune pathogenesis of viral sepsis. Front Immunol 2018; **9**: 2147.

57. Prescott HC, Girard TD. Recovery From Severe COVID-19: Leveraging the Lessons of Survival From Sepsis. *JAMA* 2020; **324.8**:739–740. doi:10.1001/jama.2020.14103

58. Beltrán-García J, Osca-Verdegal R, Pallardó FV, et al. Sepsis and Coronavirus Disease 2019: Common Features and Anti-Inflammatory Therapeutic Approaches. Crit Care Med 2020. 48(12):1841-1844

59. Kwan A, Hubank M, Rashid A, Klein N, Peters MJ. Transcriptional instability during evolving sepsis may limit biomarker based risk stratification. PLoS One 2013;8:e60501. doi:10.1371/journal.pone.0060501

60. Cazalis M-A, Lepape A, Venet F, et al. Early and dynamic changes in gene expression in septic shock patients: a genome-wide approach. Intensive Care Med Exp 2014; 2:20. doi:10.1186/s40635-014-0020-3
